# Synergistic Interactions During Co-Hydrothermal Liquefaction of Food Waste and Biomass Model Compounds for Increased Sustainable Aviation Fuel Production

**DOI:** 10.1021/acsengineeringau.6c00009

**Published:** 2026-04-27

**Authors:** Skyler B. Kauffman, Heather O. LeClerc, Alex R. Maag, Geoffrey A. Tompsett, Jeffrey R. Page, Julia A. Valla, Andrew R. Teixeira, Michael T. Timko

**Affiliations:** † Department of Chemical Engineering, 8718 Worcester Polytechnic Institute, Worcester, Massachusetts 01609, United States; ‡ Department of Chemical Engineering and Materials Science, University of Minnesota, Minneapolis, Minnesota 55455, United States; § Department of Chemical & Biomedical Engineering, University of Maine, Orono, Maine 04469, United States; ∥ Department of Chemical and Biomolecular Engineering, 7712 University of Connecticut, Storrs, Connecticut 06269, United States

**Keywords:** hydrothermal liquefaction, food waste, biomass, sustainable aviation fuel, Maillard reaction, retro-aldol condensation

## Abstract

Hydrothermal liquefaction (HTL) of lignocellulosic biomass is plagued with low biocrude yields owing to the tendency of highly reactive oxygenated intermediates to condense to form biochars. By contrast, the high protein content in food waste is comprised of substantial nitrogen species, which are known to interact strongly with oxygenates through Maillard, amide, and peptide bond formation reactions. Co-feeding food waste and lignocellulose opens new reaction pathways for biocrude formation but is currently poorly understood. This work evaluated the molecular level interactions between food waste and lignocellulose model compounds and the corresponding effect on product yields and quality. Food waste–cellulose and food waste–xylan feedstock blends achieved maximum biocrude carbon yield improvements of 12.2% and 10.1%, respectively, relative to a simple linear model that interpolates between the yields of the pure feedstocks. Increases in biocrude yield were balanced by corresponding decreases in char yield, indicating synergistic interactions between the feeds during HTL. Biocrude volatility analysis revealed that increased biocrude yield preferentially benefitted the jet fuel fraction, which comprised up to 22.6% of the total carbon yield for food waste–cellulose blends. Biocrude and char were analyzed using GC–MS and FT-IR spectroscopy to investigate the source of synergistic trends and provide greater mechanistic understanding. Key cofeeding effects included the promotion of retro-aldol condensation reactions and trans-esterification of fatty acids, sequestering carbon in the biocrude phase via the inhibition of char formation while increasing biocrude volatility toward jet fuel-range compounds. These results indicate the potential for judicious selection of HTL cofeeds to increase both biocrude yield and selectivity to desired fuel precursors, including sustainable aviation fuel.

## Graphical abstract

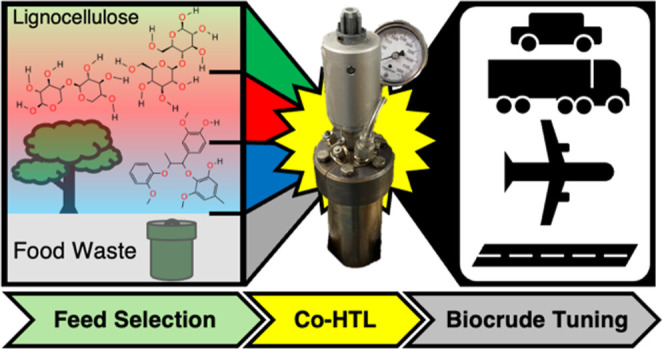

## Introduction

Greenhouse gases including CO_2_ drive global climate change,^1^ the effects of which include increased instances of severe weather events, sea level rise, ocean acidification, and agricultural losses.^2,3^ Transportation, including road transport, aviation, and shipping, was responsible for the greatest share of direct greenhouse gas emissions in the U.S. in 2022.^4^ While emissions from passenger vehicles may be effectively mitigated via electrification,^5^ the relatively low energy density of current battery technology prevents the electrification of aviation.^6^ Therefore, energy-dense and sustainable alternatives to petroleum-based jet fuel are needed to achieve climate goals.^6^

Several processes have been explored to produce sustainable fuels, including pyrolysis,^7,8^ anaerobic digestion,^9^ and hydrothermal liquefaction (HTL).^10,11^ HTL is an emerging technology capable of converting wet organic feedstocks, such as inexpensive lignocellulosic biomass or food waste, into valuable fuels or chemical precursors.^12,13^ HTL is particularly attractive due to its versatility,^11^ scalability,^14^ product quality and stability,^15^ and ability to handle high-moisture feedstocks.^10^ Typical HTL reaction conditions are between 250 and 375 °C and 10–25 MPa, generally below the critical point of water (373.9 °C, 22.1 MPa).^10,11^ At these conditions, the ionic product of water is increased relative to its familiar value at room temperature and its dielectric constant is decreased, thereby promoting some reaction rates and pathways such as acid-catalyzed reactions.^16^ The products of HTL generally include a petroleum-like biocrude, an organic-rich aqueous phase, a carbonaceous solid residue (char), and a gaseous phase.^10^ The biocrude product of HTL can be hydrotreated to produce a range of different fuels, with jet fuel (kerosene) and diesel being the most abundant light products.^17,18^ Substantial recent effort has been placed on increasing the jet fuel yield obtained from HTL as part of a new route to produce sustainable aviation fuel (SAF).^15^

The yield and properties of HTL biocrude are sensitive to both reaction conditions and feedstock composition, the effects of which have been studied previously.^19–30^ The majority of HTL research thus far has considered a single feedstock, though a growing number of recent studies have demonstrated that combining feedstocks in cohydrothermal liquefaction (co-HTL) can synergistically increase biocrude yield and alter biocrude properties.^13,31–39^ In particular, lignocellulosic biomass and food waste have emerged as promising HTL feedstocks due to their reaction synergies and favorable economic factors. The economic feasibility of HTL is tied closely to feed transportation costs, biocrude yield, and biocrude quality.^40,41^ Co-HTL has the potential to improve all three of these factors simultaneously: utilization of colocated feeds may reduce feed transportation while allowing for the tuning of biocrude properties.^42^ For example, Teri et al. observed that binary mixtures of cornstarch-soy protein and cellulose-albumin produced increased biocrude mass yields compared to the mass-averaged yield of the individual feedstocks.^36^ Additionally, Yang et al. reported a 20.9% increase in biocrude yield and simultaneous reduction in biocrude viscosity achieved by mixing spent coffee grounds and cornstalk.^31^ Déniel et al. investigated the composition of biocrudes produced from mixtures of a range of HTL model compounds, identifying coupling reactions between carbohydrates and amino acids, also known as Maillard Reactions, as a primary source of biocrude yield synergy.^33^ LeClerc et al. proposed a model consisting of elementary reactions to explain synergistic effects of Maillard reactions.^43^ In addition to the known Maillard pathway, an experimental study by LeClerc et al. observed the formation of fatty acid methyl esters (FAMEs) from co-HTL of mixtures of real food waste and green waste.^13^ While many studies point to synergistic interactions during co-HTL of multiple feeds, some studies report antagonistic effects that depress biocrude yields.^13,33,35^ Accordingly, fundamental insight is required to understand feedstock selection and maximize synergy while avoiding antagonism.

While biocrude yield is an extremely important variable determining HTL economic feasibility, biocrude yield cannot be optimized at the expense of biocrude quality. In particular, biocrude with a H/C ratio approaching 2:1 with low oxygen and nitrogen content is highly desirable to minimize the burden on downstream hydrotreating processes to produce drop-in fuels.^44,45^ In addition, the volatility range of the biocrude is extremely important for its eventual use.^18^ Residue and vacuum gas oil range products (boiling point > 340 °C) are generally most abundant in HTL biocrude, with gasoline (boiling point < 190 °C), diesel (boiling point 290–340 °C), and jet fuel or kerosene range products (boiling point 190–290 °C) also being reported.^17,18,45^ Ideally, to offset the immense carbon requirement of aviation, HTL feedstocks and conditions can be optimized for the production of SAF.^6^ In fact, HTL has been proposed as a new route to SAF, potentially offering a more economical, sustainable, and scalable route than the current method of hydrotreating vegetable oil.^6,15^ Unfortunately, information on the effects of cofed HTL on biocrude quality is currently sparse, preventing rational design of co-HTL to achieve optimal biocrude properties.

This work seeks to fill two gaps in current understanding: (1) synergistic interactions between food waste and components of biomass during HTL to boost biocrude yields, and (2) the impacts of these interactions on biocrude quality, especially for production of SAF. Biomass consists of three main biopolymers: cellulose, hemicellulose, and lignin. Each of these can potentially contribute to synergistic interactions with food waste in different ways and studying each one in isolation can provide insight into biomass feedstock selection. The present study aims to understand the interactions between food waste and lignocellulosic biomass via co-HTL of a real food waste with model compounds representing the different biopolymers present in biomass.^46^ Herein, we demonstrate that each model compound has distinct impacts on the co-HTL reaction, advancing understanding of co-HTL pathways. Furthermore, these interactions may be tailored to enhance biocrude yield and properties toward production of SAF.

## Materials & Methods

### Materials

Preconsumer prison kitchen food waste (PKFW) sourced from Coyote Ridge correctional facility (Connell, WA) was autoclaved and stored below 0 °C until use. The moisture content of the PKFW was determined by measuring the mass loss of a sample of PKFW placed in a desiccator at room temperature until a constant mass was achieved. All mass loss over this drying period was attributed to moisture, which comprised 75.25% of the total mass of the sample. The lipid and ash contents were determined using the Folch method^47^ and ASTM E1755–01,^48^ respectively. Protein content was estimated by multiplying the elemental nitrogen content of dry-basis PKFW by the standard conversion factor of 6.25.^49^ The remainder of PKFW mass was attributed to carbohydrates. The full biochemical content of the food waste used in this study is given in (Table SI1).

Lignin was derived from cosolvent enhanced lignocellulosic fractionation (CELF) of green waste sourced from Athens Services (City of Industry, CA). CELF was conducted at the University of California, Riverside, in a process which has been previously described.^50^ Avicel PH-101 microcrystalline cellulose with an average particle size of 50 μm was purchased from MilliporeSigma (Burlington, MA). Xylan from corn core purchased from TCI (Portland, OR) was used as a model hemicellulose, in accordance with prior studies.^34,51^ Full elemental compositions of all feeds are given in the (Table SI2). Deionized water with a resistance of at least 18.0 MΩ was used to fix the solids loading of each reaction at 15 wt %. ACS grade acetone (Fisher) was used for product extraction and reactor cleaning. All reagents were used as received.

### Hydrothermal Liquefaction Experiments

All HTL reactions were carried out at 300 °C with a residence time of 60 min for consistency with prior coliquefaction studies.^13,33,37,39^ Each reaction involved 22.5 g of dry organic feed and 127.5 g of water, which were loaded into a Parr benchtop reactor with 620 mL internal volume and equipped with a magnetic stirring drive and type K thermocouple. Reactions involving lignin were performed in a reactor with an internal volume of 400 mL. Before each reaction, the reactor headspace was purged three times with nitrogen gas to remove air then filled to an initial pressure of 1.38 MPa using N_2_. The reactor was heated to the operating temperature of 300 °C at a rate of approximately 7 °C/min. Once the operating temperature was reached, the reactor temperature was maintained at 300 ± 3 °C for 60 min, wherein the reactor reached peak pressures between 9.4 and 11.4 MPa, sufficient to maintain water in a near-liquid state. After the desired reaction time, the heater was switched off then the reactor was partially submerged in an ice bath for approximately 10 min to quench the reaction.

Four distinct product phases are produced by HTL: a gas phase primarily composed of CO_2_;^36,52^ an aqueous phase containing the process water, water-soluble minerals, and small water-soluble organics; a solid phase containing inorganics and hydrochar;^10^ and an oily water-insoluble biocrude. After quenching, the impeller was switched off and the internal pressure and temperature of the reactor were recorded, allowing for the calculation of the gas yield using the ideal gas law. The reactor headspace was then vented into a fume hood. The contents of the reactor were subjected to vacuum filtration to separate the aqueous phase from the solid and biocrude products. Biocrude was extracted by washing the resultant residue and the reactor walls with approximately 1 L of acetone into a second vacuum flask. The remaining acetone-washed filter cake in the funnel was dried at 60 °C for 24 h and defined as the solid product. The filtrate, a solution of biocrude and acetone, was subjected to rotary evaporation to isolate the biocrude. All runs were completed in at least duplicate, wherein all mass balances closed to >90%, and all carbon balances closed to >85%. A total of 13 different feed compositions were tested, including the four pure feeds (PKFW, cellulose, xylan, and lignin) and three mixed combinations (25:75, 50:50, and 75:25) of PKFW with each model compound. Sample naming followed a consistent convention to denote the weight fraction of the food waste and biomass model compound. Food waste content is listed first in this convention, followed by the content of the biomass model compound. For example, a sample derived from 75 wt % food waste and 25% cellulose is termed 75:25.

### Feedstock and Product Analysis

The elemental carbon, nitrogen, and hydrogen (CHN) contents of all feeds, biocrudes, and dried solid products were determined via commercial elemental analysis by either Midwest Microlabs (Indianapolis, IN) or Microanalysis Inc. (Wilmington, DE). Oxygen content was calculated by difference. The organic carbon content of the aqueous phase was determined by total organic content (TOC) analysis using a Shimadzu TOC-L series analyzer. The carbon content of the gas was estimated by attributing all gas evolution to CO_2_ formation.^53^ The quantity of dissolved gas in the liquid phase was assumed to be negligible. The total carbon content of biocrude and char products was determined by multiplying the gravimetrically determined mass yield by elemental carbon content. Carbon yield of aqueous products was estimated based on the mass of the water product and its TOC. Carbon yield was defined as the total carbon content of a given product phase divided by the total carbon content of the organic feed.

Thermogravimetric analysis (TGA) was used to determine the boiling point distribution of biocrude samples. A Shimadzu TGA-50 was used for all TG measurements, using a method similar to Zhang et al.^52^ Samples were first heated to 110 °C to eliminate moisture, then heated to 800 °C at a constant rate of 5 °C/min in an inert atmosphere maintained by a flow of 25 mL/min of nitrogen gas. Analysis was performed for one biocrude sample per feed condition. Distillation cut temperatures were defined according to Haider et al.^17^ Fuel fractional mass yields were defined according to accumulated sample mass loss between distillation cut temperatures. Cheng et al. report the thermal degradation of a similar HTL biocrude at temperatures exceeding 410 °C, so the quantification of the high boiling point fractions may include mass loss due to thermal cracking of large compounds.^54,55^ Fuel fractional mass yields were multiplied by biocrude carbon yields to estimate the carbon yield for each fraction of each biocrude. Compounds with boiling points between 100 and 350 °C have a carbon content similar to that of a bulk HTL biocrude,^18^ making this a reasonable estimation.

Gas chromatography with mass spectrometry (GC–MS) was used to investigate the molecular compositions of all biocrude and aqueous phase products. Biocrudes were diluted in HPLC grade methanol (Fisher) and filtered to 0.22 μm prior to injection. Aqueous phase samples were acidified to a pH of 3.5 or lower using 1 N hydrochloric acid (Fisher), then mixed with an equivalent volume of HPLC grade dichloromethane to extract aqueous organic components. Acidification of the aqueous phase was used to enhance the partitioning of organics into the nonpolar DCM phase.^56^

All GC–MS analysis was conducted using a Shimadzu GC-2010 Plus gas chromatograph (GC) equipped with a 30 m Restek RTX-5 column coupled to a Shimadzu QP2010 SE MS quadrupole mass spectrometer (MS). Helium (UHP Airgas) was used as the carrier gas, with a total flow rate of 9.8 mL/min. The GC was equilibrated at 35 °C for 4 min, then ramped at a rate of 5 °C/min to 290 °C, at which point the temperature was maintained for 40 min. All molecular assignments were made using the NIST library.

GC–MS analysis is an effective method to characterize only compounds with boiling points below 300 °C,^57^ necessitating the use of additional characterization methods for the less volatile fraction. Fourier transform infrared (FT-IR) spectroscopy was used to characterize the functional groups of all biocrude and char samples. A PerkinElmer Spectrum Two with a PerkinElmer UATR Two attenuated total reflectance attachment was used for all measurements. Spectra were accumulated from 32 scans over the range 4000 cm^–1^ to 400 cm^–1^ with a resolution of 4 cm^–1^. Band assignments were made according to various literature sources.^58–68^

## Results & Discussion

This study aims to understand the synergistic and antagonistic behavior reported in previous studies on cohydrothermal liquefaction^13,31,33,35^ through the experimental analysis of individual green waste components with food waste. A realistic food waste stream, obtained from a cafeteria, was used for all experiments. In addition, three green waste model compounds were chosen to represent the three primary macromolecular structures of cellulose (C), hemicellulose (X), and lignin (L), where xylan was utilized as a model for hemicellulose. Pure feeds and mixtures of food waste with each of the biomass model compounds were treated under HTL conditions to quantify yields of the biocrude, char, aqueous phase, and gas products. The biocrude, char, and aqueous phase products were then analyzed in various ways, including volatility to identify fuel fractions in the biocrude and at a molecular level using mass spectrometry and spectroscopy. These results were then integrated with one another to develop insight into synergistic and antagonistic interactions that occur during HTL and their corresponding effects on biocrude quality.

### Hydrothermal Liquefaction Product Yields

The starting point for this study was quantification of product yields from co-HTL of food waste and biomass model compounds. Gravimetrically determined carbon yields are shown in Figure 1, with numerical values provided in Table SI3 in Supporting Information. Numerical values for product mass yields and biocrude elemental content are provided in Table SI4. In most cases and for both pure and mixed feeds, biocrude and char were the two most abundant products, followed by aqueous, and finally gas-phase products. Considering pure feeds more closely, yields from pure cellulose^35,36,69^ and pure food waste,^13,30,70^ (14.5% and 49.0%, respectively) were comparable to previous HTL studies using similar feeds and reaction conditions. Further, the food waste biocrude yield was approximately three times greater than the biocrude yield obtained from HTL of any of the three biomass model compounds. This effect is likely attributable to the direct hydrolysis of lipids (which comprise 18.6 wt % of the food waste) to fatty acids, which primarily partition into the biocrude.^35,71^ HTL of lignin and carbohydrates such as cellulose and xylan, on the other hand, has been shown to produce a wide range of water-soluble aldehydes, alcohols, ketones, and phenols which are prone to repolymerization and char formation.^69,72,73^ The specific product formation pathways associated with each feed will be discussed in greater detail in the following sections. Thus, biocrude yields generally decreased with increasing model biomass compound content in the feed while char yields increased with increasing model compound content. Gas and aqueous phase yields were weaker functions of composition than either biocrude or char yield.

**Figure 1:**
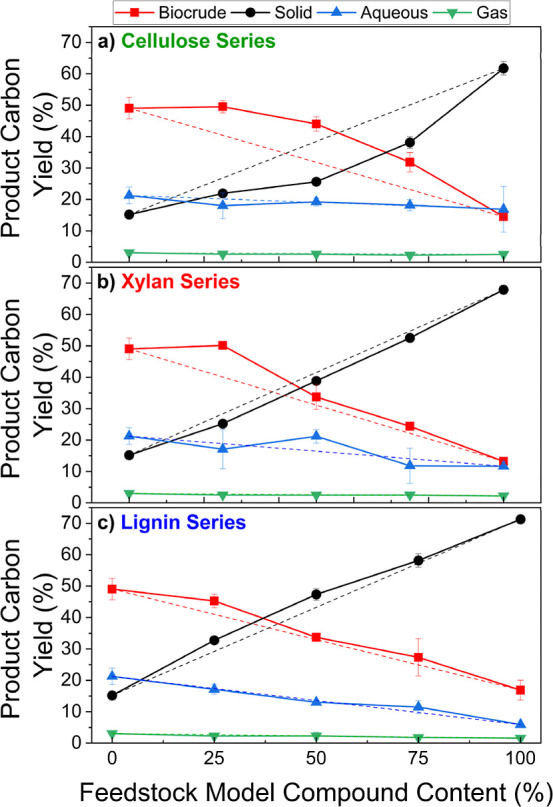
Carbon yields of all product phases for mixtures of food waste and (a) cellulose, (b) xylan, and (c) lignin following HTL reactions at 300 °C with a residence time of 60 min, calculated relative to the total carbon content of the organic feed. Dashed lines represent the biocrude yields predicted using a simple linear model based on the yields observed for pure components. Deviation from the linear interpolations may be attributed to emergent chemistry. Error bars represent the experimental standard deviation calculated from runs performed in at least duplicate.

Figure 1 further reveals synergistic and antagonistic trends in product yields as assessed by deviation of measured yields from those predicted by a simple linear model based on the weighted contributions of the two corresponding pure feeds. Product yields exhibited distinct maxima and minima that contrast with the monotonic predictions of the linear model, wherein the difference between the data and linear model represents the magnitude of synergistic (positive deviation) or antagonistic (negative deviation) effects on co-HTL yields. Co-HTL of the 50:50 food waste–cellulose blend resulted in the greatest synergistic effect (increased biocrude), observed in this study. Relative to the linear model, biocrude yield was 12.2% greater than expected, while char yield was 12.8% lower than expected for this feed composition. The highest total biocrude yields were observed for the 75:25 food waste–cellulose (49.5%) and 75:25 food waste–xylan blends (50.2%), both slightly exceeding that of pure food waste (49.0%) and representing synergistic improvements of 9.1% and 10.1%, respectively, relative to the linear models. Blends of food waste with lignin exhibited much milder effects compared to the cellulose and xylan series. Only the 75:25 food waste–lignin blend exhibited a notable synergistic effect (3.2% improvement).

Interestingly and unlike some previous studies, biocrude yield antagonism is not observed in the current study.^13,31,35^ For example, LeClerc et al. observed a decrease in biocrude yield (relative to the mass average-weighted value) for blends of 25% food waste and 75% lignocellulosic biomass.^13^ The difference between the current work, which did not uncover evidence of antagonistic effects on biocrude yield, and previous reports which include antagonistic interactions can likely be attributable either to third-order effects (e.g., interactions between food waste and two model compounds simultaneously) or possibly to compositional differences between feeds. The model lignocellulose compounds used in this study were effectively devoid of ash (<1 wt %), whereas whole green waste and biomass streams consist of between 4.3 and 18.6 wt % ash.^46^ The food waste used in this study was not devoid of ash, containing 5.8% ash by mass. Among other effects, food waste ash can have an alkali character,^74^ hence modulating relevant biocrude formation pathways. Decomposition of amino acids may also lead to formation of ammonia, raising aqueous phase pH and potentially altering product yields, compositions, and formation pathways.^69,75^ By using a fixed food waste composition and biomass model compounds with low ash content, the current study isolates the effects of the biopolymers themselves from potential effects of feedstock ash, revealing much more subtle interactions than can be observed for whole biomass or green waste.

### Biocrude Elemental Analysis

Having quantified synergistic effects leading to increased biocrude for co-HTL of food waste with both cellulose and xylan, the next step was biocrude analysis. The starting point for biocrude analysis was elemental composition, provided both in Table SI4 and as a plot of H–C ratio in Figure SI1. A major goal of biofuel production is a H–C ratio greater than 1.5, since petroleum-derived fuels exhibit H–C ratios of approximately 1.6 to 1.9.^76^ The biocrude obtained from HTL of 100% food waste had a relatively high H–C ratio of 1.7, higher than biocrude obtained from any of the model compounds and likely attributable to the high fatty acid content of food waste. The H–C ratios of biocrudes from pure cellulose (1.3), xylan (0.9), and lignin (1.3) were much lower than the food waste biocrude, reflecting their high composition of aromatic and oxygenated compounds.^13,35^ Most strikingly, co-HTL of food waste and xylan produced biocrudes with systematically greater H–C ratio than expected from linear trends. Co-HTL of food waste and cellulose mainly resulted in biocrude with H–C ratio similar to that of pure cellulose biocrude, representing an antagonism compared to linear interpolation. The H–C ratio of biocrudes from co-HTL of lignin and food waste generally followed linear trends aside from the 25:75 ratio, which resulted in a higher-than-expected H–C ratio.

### Biocrude Volatility Range Analysis

Following elemental analysis, the next property considered in this study was volatility, a key biocrude property that affects its suitability for different applications and production for different types of fuels.^18,77^ In particular, volatility provides insight into the likely yields obtained from hydrotreating biocrude, with desired products exhibiting volatility similar to gasoline, diesel, or jet fuel and less desirable products exhibiting lower volatility. Historically, distillation of petroleum is used to quantify its distribution into fractions based on volatility, following ASTM D86.^78,79^ Precise fractionation using laboratory distillation equipment requires larger sample volumes than is practical.^78^ Thermogravimetric analysis (TGA) is an accepted method of quantifying oil volatility, and has been found to yield similar results as obtained using laboratory distillation.^80–82^ Accordingly, biocrude volatility was estimated using TGA (Figure SI2) and is represented by differential thermograms (DTG) highlighting mass loss as a function of temperature (Figure SI3). In summary, TGA measurements provide a qualitative indication of shifts in volatility that are relevant for downstream applications.^83^

The raw data in Figure SI3 were integrated to quantify the yield of gasoline, jet fuel (kerosene), diesel, vacuum gas oil, and residue fractions, as shown in Table SI5 and plotted in Figure 2. It should be noted that most of the compounds in the biocrude may not be suitable for direct use as fuels due to high oxygen and nitrogen content, thus requiring further upgrading and rendering the assignment to crude oil fractions illustrative rather than prescriptive. That stated, common biocrude upgrading technologies, such as hydrodeoxygenation, involve the liberation of oxygen from biocrude compounds in the form of water, preserving the underlying carbon backbone as an alkane and moderately increasing volatility.^45,84^ Therefore, systematic volatility shifts in the unprocessed biocrude would likely be preserved in the final, upgraded product with an increased bias toward lighter gasoline-range and jet fuel-range compounds.

**Figure 2:**
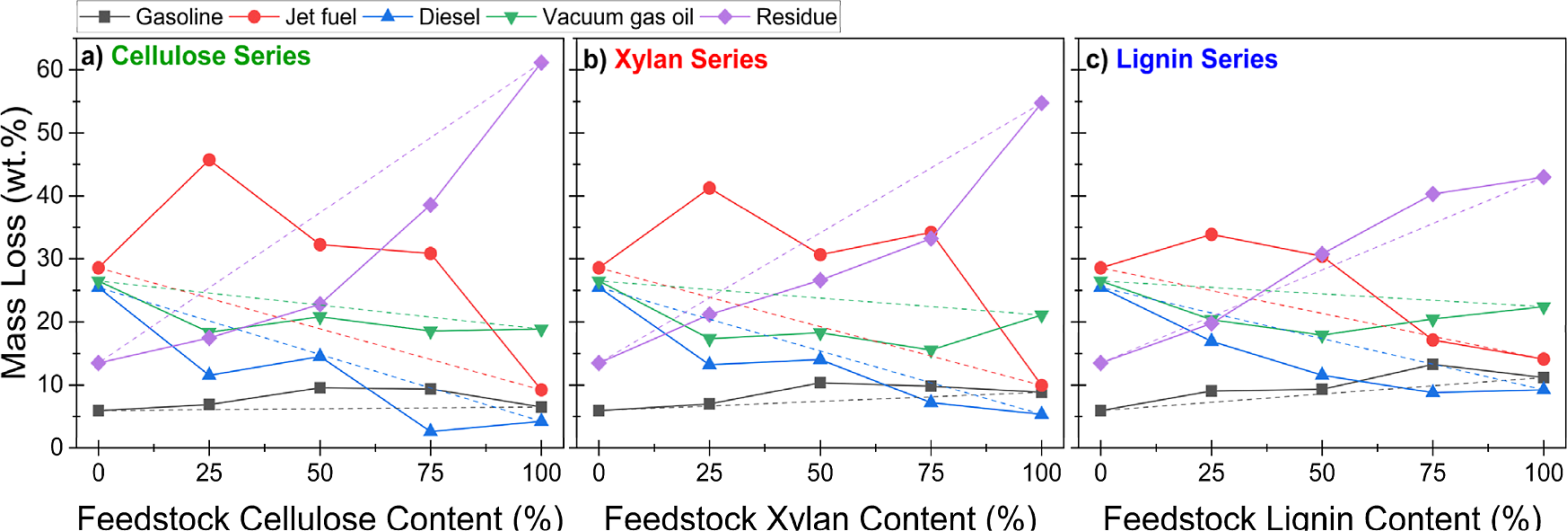
Trends of simulated fuel fractions for each HTL biocrude as a function of feedstock model compound composition compared to the expected linear trend for blends of food waste with (a) cellulose, (b) xylan, and (c) lignin, determined from TGA by integrated mass loss over defined temperature ranges. Temperature ranges are gasoline ≤ 190 °C, jet fuel = 190–290 °C, diesel = 290–340 °C, vacuum gas oil = 340–538 °C, residue ≥538 °C.^17^ Uncertainties are discussed in the methods section.

Co-HTL of food waste and green waste model compound mixtures increased the low boiling point (high-volatility) fractions and decreased high boiling point (low-volatility) fractions for all three model compound mixtures (Figure 2). Given recent interest in low cost production of SAF,^6^ the maximum jet fuel fraction (defined as integrated mass loss between 190 and 290 °C) was obtained at 75% food waste and 25% model compound for all three model compounds explored in this study, with mass yields of 45.7%, 41.3%, and 9.1% for cellulose, xylan, and lignin blends, respectively, corresponding to increases of 22.0%, 17.3%, and 8.9% relative to linear interpolation between the pure feeds. Over the same range of feed ratios, the mass yields of the undesirable heavier vacuum gas oil (VGO) and residue fractions were often suppressed. For example, the 22.0% increase in jet fuel mass yield observed for the 75:25 food waste - cellulose blend was accompanied by a 6.2% decrease in VGO yield and a 7.9% decrease in residue yield. This finding indicates that the ratio of food and green waste may be a tunable metric in predicting the distillation range of biocrude acquired from HTL. Accordingly, Figure 2 highlights a very encouraging result that co-HTL can result in controlled production of a targeted fuel range, in addition to the well-documented impact of co-HTL on biocrude yields.^13,31,35^

For co-HTL of food waste and all three model compounds, the yield of vacuum residue increased monotonically with model compound content, reaching a maximum for HTL of the pure model compound. Pure food waste yielded only 13.5% residue, while residue mass yields were 61.2%, 54.7%, and 43.0% for pure cellulose, xylan, and lignin, respectively. Low volatility of lignocellulose biocrude is a known hurdle for sustainable fuel production from HTL.^77,85^ For example, Yu et al. reported that more than 60% of a biocrude produced from aspen wood boiled above 340 °C.^85^ On the other end of the volatility range, gasoline yields were either approximately constant with respect to composition (e.g., cellulose co-HTL with food waste, Figure 2a) weakly increased with model compound content (Figure 2b,c). In all cases, gasoline mass yields were less than 15%. Diesel (25.5%) and VGO (26.5%) are maximized for HTL of pure food waste, decreasing with increasing co-HTL of model green waste compounds. These observations indicate synergistic interactions, particularly at the 75:25 feed composition ratio, a finding which merits greater scrutiny.

Combining the carbon yields (Figure 1) with the TGA fuel fractions (Figure 2) enables detailed analysis of the relative carbon yields of particular fuel ranges that can be attained through co-HTL (Figure 3), providing a quantitative representation of total fuel yields relative to the feed. The jet fuel range revealed strong synergistic effects with all three green waste model compounds at nearly all feedstock blends due to the compounding effect of increases in both total biocrude yield and jet fuel mass fraction for food waste–model compound blends. Jet fuel carbon yield was maximized at the 75:25 feed ratio for all three series, with the highest total yield observed for the 75:25 blend of food waste with cellulose (22.6%), followed by the xylan (20.7%) and lignin (15.0%) blends (Figure 3). The 75:25 food waste–cellulose blend also yielded the greatest synergistic improvement, outperforming the linear interpolation by 11.8%. In other terms, this feed blend yielded more than double the expected quantity of carbon in the jet fuel range. While these improvements do correspond to a small decrease in diesel yield consistent with the shift to higher volatility products (Figure 2), this reduction is not sufficient to explain the entirety of the jet fuel synergy (Figure 3), further suggesting the presence of additional synergistic interactions. For example, while the 75:25 food waste–cellulose blend resulted in a jet fuel yield 11.8% greater than expected, the diesel carbon yield was only suppressed by 3.8%. Similar behavior was observed across the range of feed compositions for all three series. Generally, mild antagonism was observed for VGO and mild synergy was observed for the residue fraction, which largely cancel when added together as the heavy fraction. Although lower in abundance (and, as such, subject to greater uncertainty), the gasoline fraction also exhibited notable synergistic improvements of approximately 1%–2% across all feed blends. Individual yield plots for all fuel ranges can be found in Figure SI4. Given that the future of transportation requires sustainable sources of jet fuel and gasoline,^86,87^ tunable production of these important fuel ranges opens compelling new opportunities for co-HTL.

**Figure 3:**
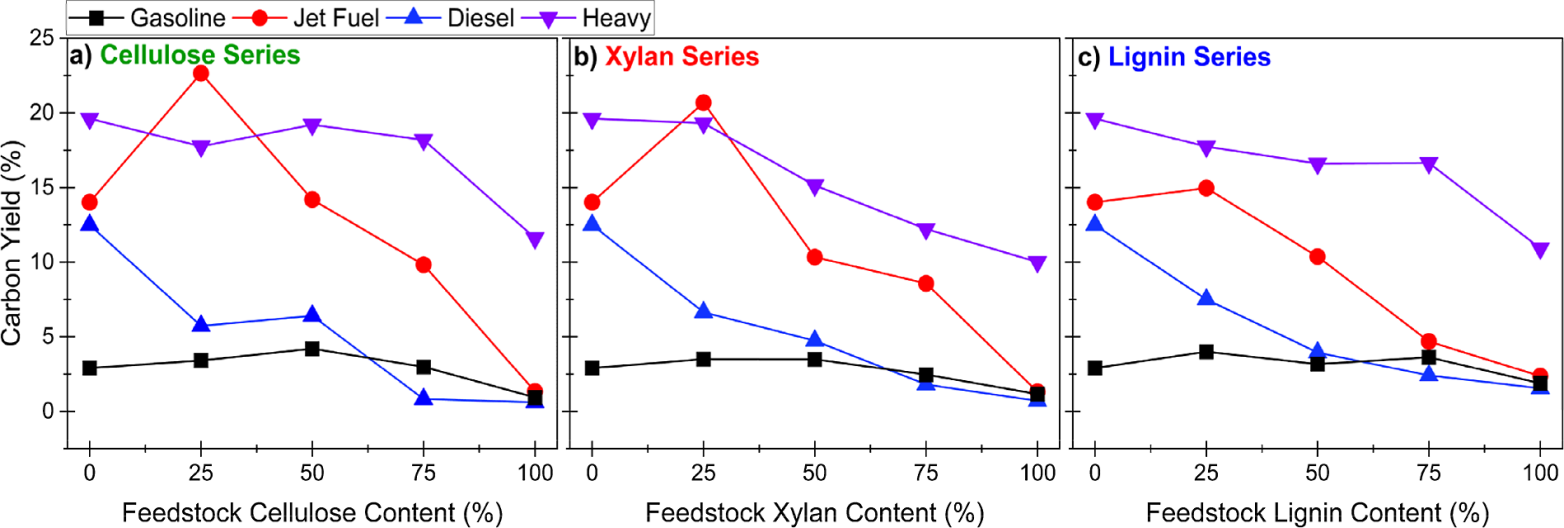
Carbon yield-weighted simulated fuel fractions compared to the theoretical linear trend for (a) cellulose series, (b) xylan series, and (c) lignin series biocrudes, calculated relative to the total carbon content of the organic feed. The low-volatility heavy fraction is the sum of VGO and residue (≥340 °C). Uncertainties discussed in the methods section.

### Molecular Analysis by GC–MS

Identification of synergy in co-HTL biocrude yields and transportation fuel composition motivates additional molecular-level analysis to further understand the behavior. To investigate the molecular changes occurring during co-HTL, biocrudes and aqueous phases was analyzed using GC–MS. Due to the volatility dependence of mixture analysis using GC–MS, only the volatile fraction (approximately gasoline, jet fuel, and diesel) of the biocrude was sufficiently analyzed for semiquantitative determination of specific molecular components (Figure SI5). Additional characterization of small water-soluble products is provided by GC–MS analysis of the aqueous phase (Figure SI6). Integrated areas of key peaks identified in biocrude GC–MS chromatograms are shown in Figure 4; Table SI6, located in the Supporting Information, provides a list of peak assignments. To facilitate analysis, identified compounds were grouped into amides, fatty acid methyl esters (FAMEs), fatty acids, aromatics, furanics, phenols, and other oxygenates for intercomparison. Superficially, HTL of pure food waste resulted in a biocrude with the largest fraction of fatty acids, consistent with direct triglyceride hydrolysis of the high lipid content of the food waste (18.6 wt %).^35,71,88^ Other findings of the products of HTL of pure feeds are similarly consistent with expectation, e.g., high phenol yield obtained from HTL of pure lignin. Closer consideration of the product distribution was then performed to identify molecular evidence of both synergistic and antagonistic interactions.

**Figure 4:**
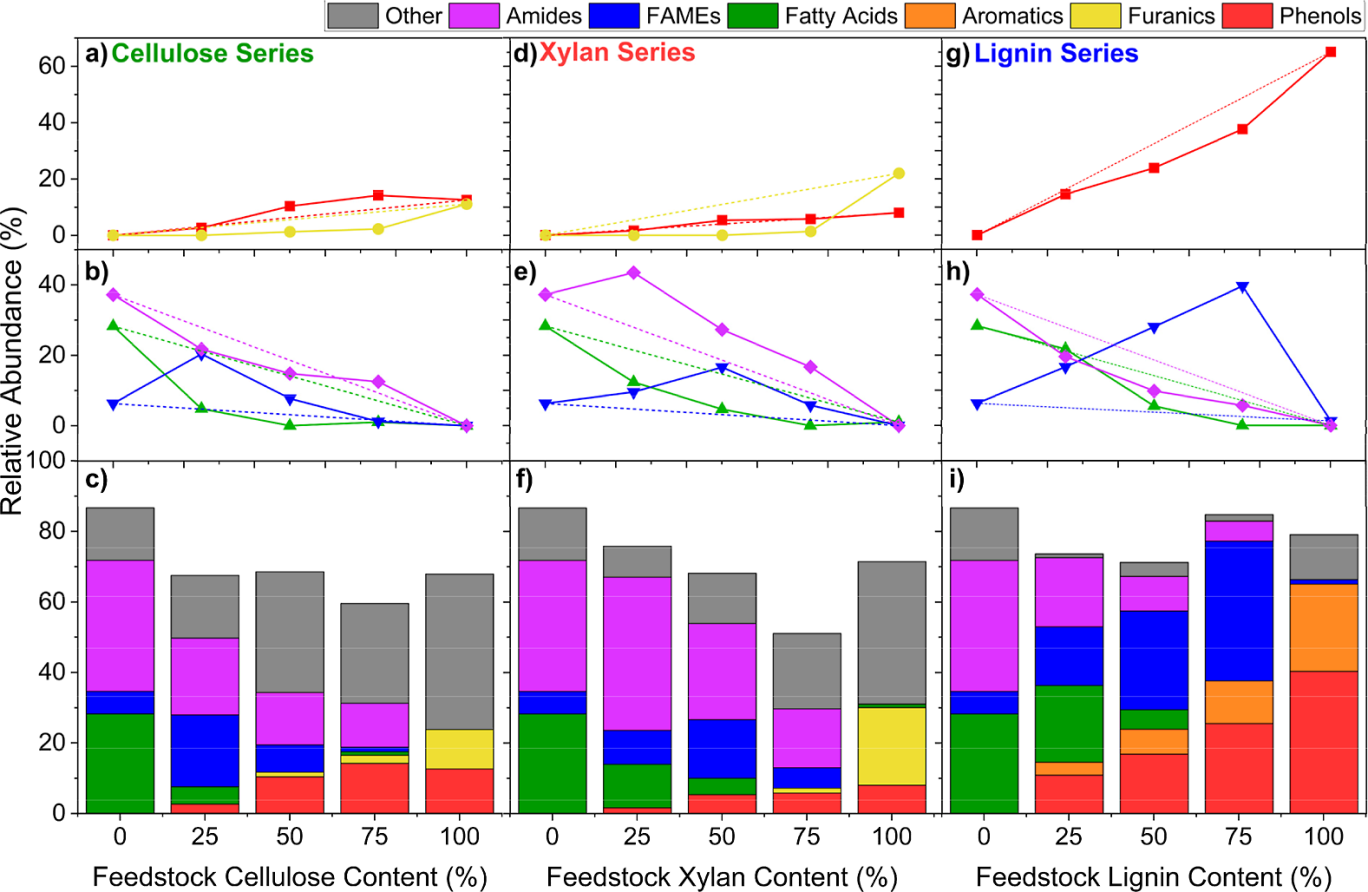
Relative abundance of compound classes identified by GC–MS in all (a–c) cellulose series, (d–f) xylan series, and (g–i) lignin series biocrudes. Individual peaks were integrated to obtain their area and relative abundance within each chromatogram. Compounds with peaks representing ≥1% of total peak area are included for analysis.

Starting first with the food waste-cellulose series, the FAME fraction was greatest in the 75:25 food waste–cellulose biocrude, accounting for 20.4% of the total abundance compared with 6.3% in the pure food waste biocrude (Figure 4a–c) which is a 14.1% increase. Synergistic production of FAME indicates an important synergy exists associated with fatty acid esterification with methanol liberated from small oxygenates.^56,71^ FAME production is coupled with a sharp decline in fatty acid abundance (28.3% for pure food waste to 4.9% for 75:25 food waste–cellulose), further confirming the esterification hypothesis. The shift from fatty acids to FAMEs is consistent with the volatility shift observed for the food waste–cellulose biocrudes (Figure 2), wherein the primary mass loss peak in the pure food waste DTG curve (Figure SI3) centered at ∼300 °C and corresponding to fatty acids^45^ shifts to a lower temperature within the jet fuel range. The FAMEs have lower boiling points than their fatty acid counterparts^89^ leading to increased volatility and a jet-fuel enriched biocrude. Fatty amides, which result from reaction of fatty acids with amino acid breakdown products, are maximized for pure food waste (37.2%) and decrease linearly with decreasing food waste content to 0% for pure cellulose, consistent with a simple reaction determined by the availability of the two reactants. Similarly to fatty amides, phenols and furanics showed minimal emergent behavior, although phenolic content reached a maximum of 14.2% with a 75% cellulose feedstock, representing a approximately 50% increase relative to linear interpolation, indicative of enhanced glucose dehydration reactions.^90^ The furanics were found to be most abundant in the pure cellulose biocrude (11.2%) which decreased sharply between pure cellulose and 75% cellulose biocrude (2.3%).

Considering xylan, co-HTL of food waste and xylan (Figure 4d–f), FAME production was maximized at a 50:50 mixture (16.6%), a distinct shift from the trend seen with cellulose. Instead, amides were more abundant than FAMEs for all feed ratios of mixed xylan and food waste, reaching a maximum abundance of 43.5% for the 25% xylan biocrude. Similarly to the cellulose series, however, the shift to higher volatility products may be attributable to trans-esterification reactions.^33,34,89^ Furfural (from dehydration of xylose) was found to be a dominant product class in pure xylan biocrude, accounting for 22.0% of GC–MS abundance which dropped to nearly undetectable levels with the addition of as little as 25% food waste.

Lastly, in the food waste-lignin series (Figure 4g–i) the most striking observation is a sharp, linear increase in FAME content from a pure food waste biocrude to a 25:75 food waste: lignin biocrude. The maximum FAME content was found in the 25:75 biocrude, wherein fatty acid content decreased to nearly 0% while FAME content reached 39.6%. The linear trend between FAME production and lignin content is consistent with liberation of methanol by side chain scission of the phenolic monomers from lignin reacting in esterification reactions with fatty acids.^13^ Notably, the increase in FAME abundance, particularly for the 75% lignin blend, exceeds the decrease in fatty acid content. It is likely that fatty acid capping via esterification reduces the potential for further condensation,^13^ sequestering a greater fraction of the fatty acids in the biocrude as FAMEs. However, the lack of biocrude yield synergy for the lignin series suggests that, if present, this mechanism is either minimal or balanced by antagonistic pathways. Due to the lack of fatty acids in pure lignin, the FAME content of the pure lignin biocrude is 0%. Instead, the phenolic compounds were most abundant in the pure lignin biocrude (65.1%) and decreased nearly linearly with decreasing lignin content to undetectable levels for pure food waste biocrude. This behavior suggests lignin decomposition as the primary phenolic source with minimal interactions with food waste breakdown products.^34,35^

Revisiting the interesting trends observed for furanics in the cellulose and xylan series, several specific types of cyclic ketones (i.e., 2-methyl,2-cyclopenten-1-one) were identified in the mixed feed biocrudes and aqueous phase extracts (Figures SI5 and SI-6). An increase in the abundance of cyclic ketones corresponded with a decrease in the abundance of 2,5-hexanedione and 5-hydroxymethylfurfural (5-HMF). This shift toward cyclic ketones is indicative of retro-aldol condensation reactions, which are a base-catalyzed reaction family.^33,91^ The strong dependence of cyclic ketone yields on food waste content further implies that food waste may be acting as a buffering agent or alkali source.^33,91^ As further support of this interpretation, selectivity between furfural and cyclopentanone in the aqueous phase is shown as a function of pH in Figure SI7, where furfural selectivity drops from 2.56 for pure xylan to 0.21 for 75% xylan. A less dramatic shift in selectivity is observed between pure cellulose (0.21) and 75% cellulose (0.12) due to the greater abundance of linear ketones than furfurals. Postreaction aqueous phase pH trends are shown in Figure 5, revealing a systematic shift toward lower pH for reactions involving increased model compound feed. For example, a large change in postreaction pH from 2.6 for pure cellulose to 5.5 for pure food waste was observed, consistent with the idea that food waste acts as an alkali catalyst, likely in the form of amines and/or ash.^74,75^

**Figure 5:**
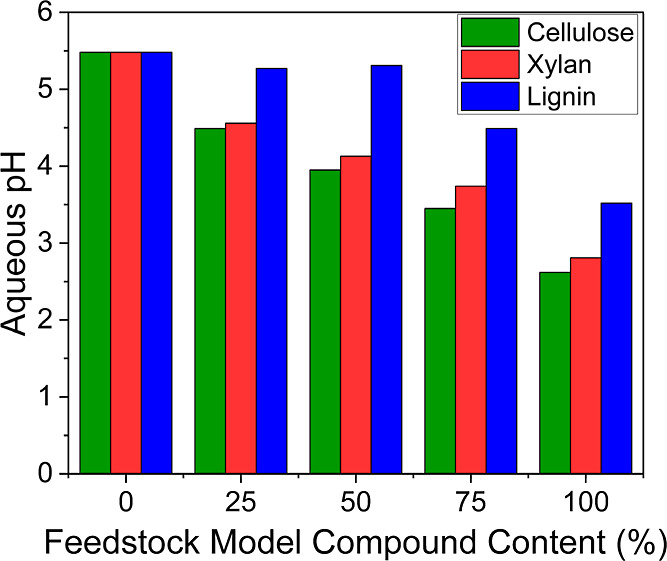
Postreaction pH for co-HTL aqueous phases following HTL processing of food waste–model compound blends at 300 °C for 60 min.

The alkali species provided by food waste and their subsequent degradation products can play additional beneficial roles in both cellulose and xylan conversion. After formation, cyclic ketones may undergo controlled polymerization with furfurals in the presence of an alkali catalyst, which result in jet fuel or diesel-range products^92^ rather than continuing to polymerize into char-phase molecules.^72^ This polymerization mechanism is further consistent with the observed increase in jet fuel range products and the corresponding decrease in char for co-HTL of food waste with cellulose and xylan (Figure 1). Furthermore, alkali catalysts are known to promote carbohydrate hydrolysis,^35,93,94^ increasing the availability of small ketones and furanic compounds for polymerization reactions toward biocrude-range molecules. Accordingly, the synergistic effects of food waste-derived alkali promoted retro-aldol condensation reactions may explain the decreased furfural content in co-HTL of cellulose and xylan.

### Functional Group Analysis by FT-IR Spectroscopy

GC–MS has the advantage that it provides information on specific product molecules. The disadvantage of GC–MS is that it cannot provide information on molecules with insufficient volatility to vaporize in the inlet or pass through the column. Operationally, this limitation means that compounds with boiling points above 300 °C typically cannot be detected using GC.^57^ As an alternative to GC, FT-IR provides functional group information for the entire product distribution, irrespective of volatility. Raw FT-IR spectra are provided in Figures SI8 and SI9. Raw spectra were analyzed to identify key bands, the peak absorbances of which were used in Figure 6 to show the effects of composition on functional groups present in co-HTL biocrude.

**Figure 6:**
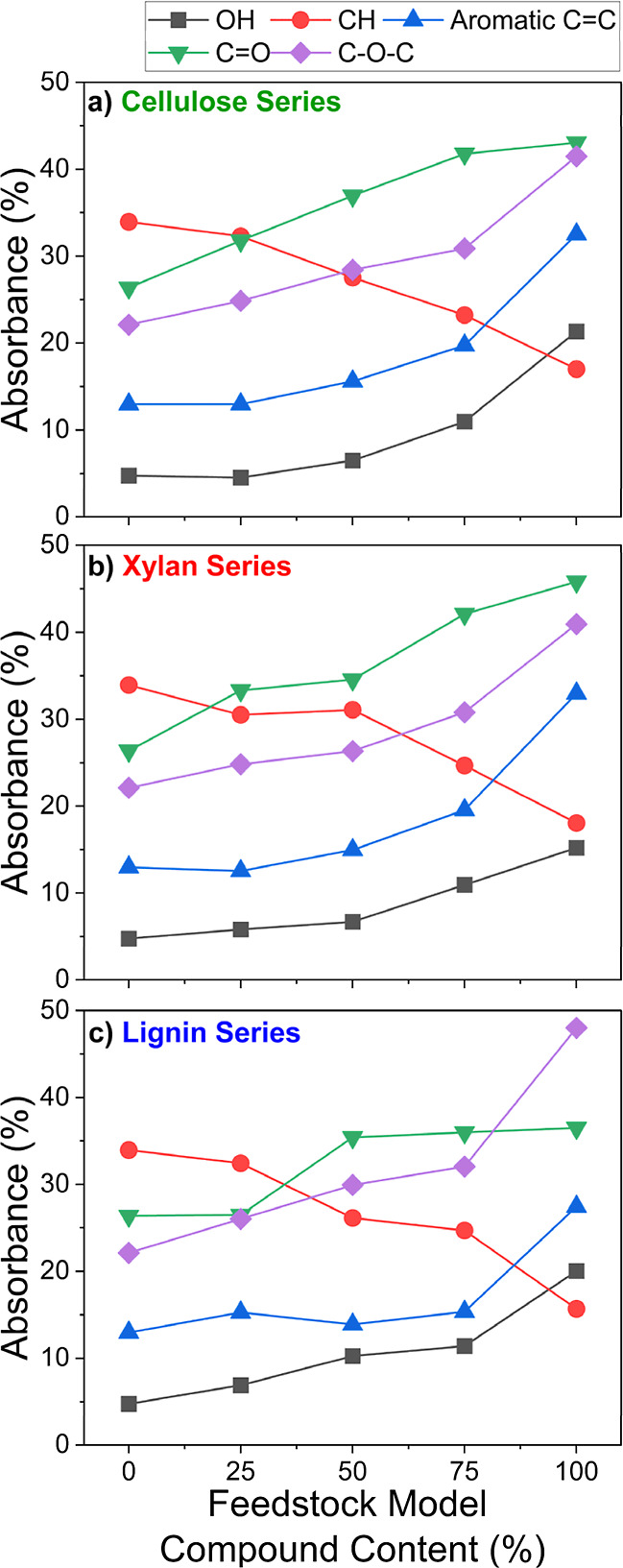
Absorbance of key FT-IR bands from (a) cellulose series, (b) xylan series, and (c) lignin series biocrudes. Raw spectra are provided in Figure SI7 in the Supporting Information.

First considering the pure feed HTL biocrudes, food waste biocrude spectra were dominated by C–H and C=O groups, consistent with the fatty acid content indicated by GC–MS.^58,59^ In the cellulose and xylan-series biocrudes, C=O, C–O–C, and C=C groups were most prevalent, consistent with the furans identified in GC–MS, potentially as well as residual glycosidic bonds from the carbohydrate biopolymers.^60,62^ Finally, the lignin spectrum is dominated by C–O–C and C=O, with C=C present in lesser abundance. Given the known aromatic content of lignin and the phenolic monomers present in its GC–MS chromatogram, Figure 6c indicates that the heavier fraction of the lignin HTL consists of oligomers with abundant ether, C–O–C, linkages, and abundant C=O side chains, consistent with observations made using high resolution mass spectrometry of lignin HTL biocrude.^73^

Considering the FT-IR spectra of mixed-feed biocrudes, several trends are apparent in Figure 6. First, the C–H band decreases monotonically with decreasing food waste content due to the decreased abundance of fatty acids. Antagonism is apparent for the aromatic C=C band for food waste mixed with any of the three biomass model compounds. Since GC analysis revealed mild cellulose and xylan synergy with food waste for phenol production, FT-IR analysis implies that the antagonistic effect on C=C bonds is due to suppressed formation of large, likely residue-range aromatic species which are not GC-analyzable, signifying inhibition of polymerization reactions.^69^ In fact, the suppressed formation of heavy compounds containing C=C groups is consistent with the suppression of char for co-HTL of food waste and biomass model compounds, shown previously in Figure 1. The heavy C=C bearing compounds present in biocrude are precursors to char,^33,72^ meaning that co-HTL suppresses both precursors and char formation. On the other hand, the pronounced aromatic C=C antagonism observed for 50% and 75% lignin is consistent with the phenolic trend identified using GC analysis, suggesting that smaller phenolic compounds drive trends observed by both GC–MS and FT-IR.

An antagonistic tendency is apparent for the ether linkage vibration across all three model compound series. For all three series, this is attributable due to decreased polymerization of intermediates toward large, residue-range molecules or enhanced depolymerization of the ether-linkage rich structure of the model compounds.^72,73^ The C–O–C band shows antagonism in all three series, particularly in the comparison between the pure model compound and 75% model compound biocrudes, provides further support for the apparent catalytic effect of food waste on carbohydrate and lignin depolymerization.

Effects of co-HTL on emergent effects on the C=O vibration are mixed. The general trend is that the C=O intensity increases with increasing model compound content. For hemicellulose and lignin, the increase is roughly linear, whereas for cellulose there is apparent synergy. The C=O vibration can arise from multiple functional groups, including aldehydes, ketones, amides, and carboxylic acids, meaning that simple synergistic and antagonistic trends are not easily attributable. That stated, synergistic formation of cyclic ketones, which are less prone to polymerization than aldehydes, is consistent with the IR spectra observed for food waste—cellulose biocrudes. Synergistic formation of carboxylic acids can be discounted since the O–H stretch exhibits antagonism between food waste and cellulose, attributable in part to the aforementioned formation of FAMEs.

### Reaction Pathway Discussion

Carbon yields, biocrude volatility, GC–MS, and FT-IR data can be combined with previous reports to form an internally consistent description of the synergistic and antagonistic interactions arising from co-HTL of food waste and different biomass model compounds. Key pathways that are implicated by this work are summarized in Figure 7, emphasizing interactions of the lipid, biomass carbohydrate, and lignin fractions with one another. Key initial steps include hydrolysis of triglycerides present in food waste to produce fatty acids.^35^ In parallel, HTL of lignin, and to a lesser extent cellulose and xylan, produces methanol, which then undergoes trans-esterification reactions with diesel range fatty acids to form jet fuel range FAMEs in the mixed-feed reactions.^13^ This reaction scheme decreases the volatility of compounds within the biocrude, benefiting the jet fuel fraction at the expense of diesel.

**Figure 7:**
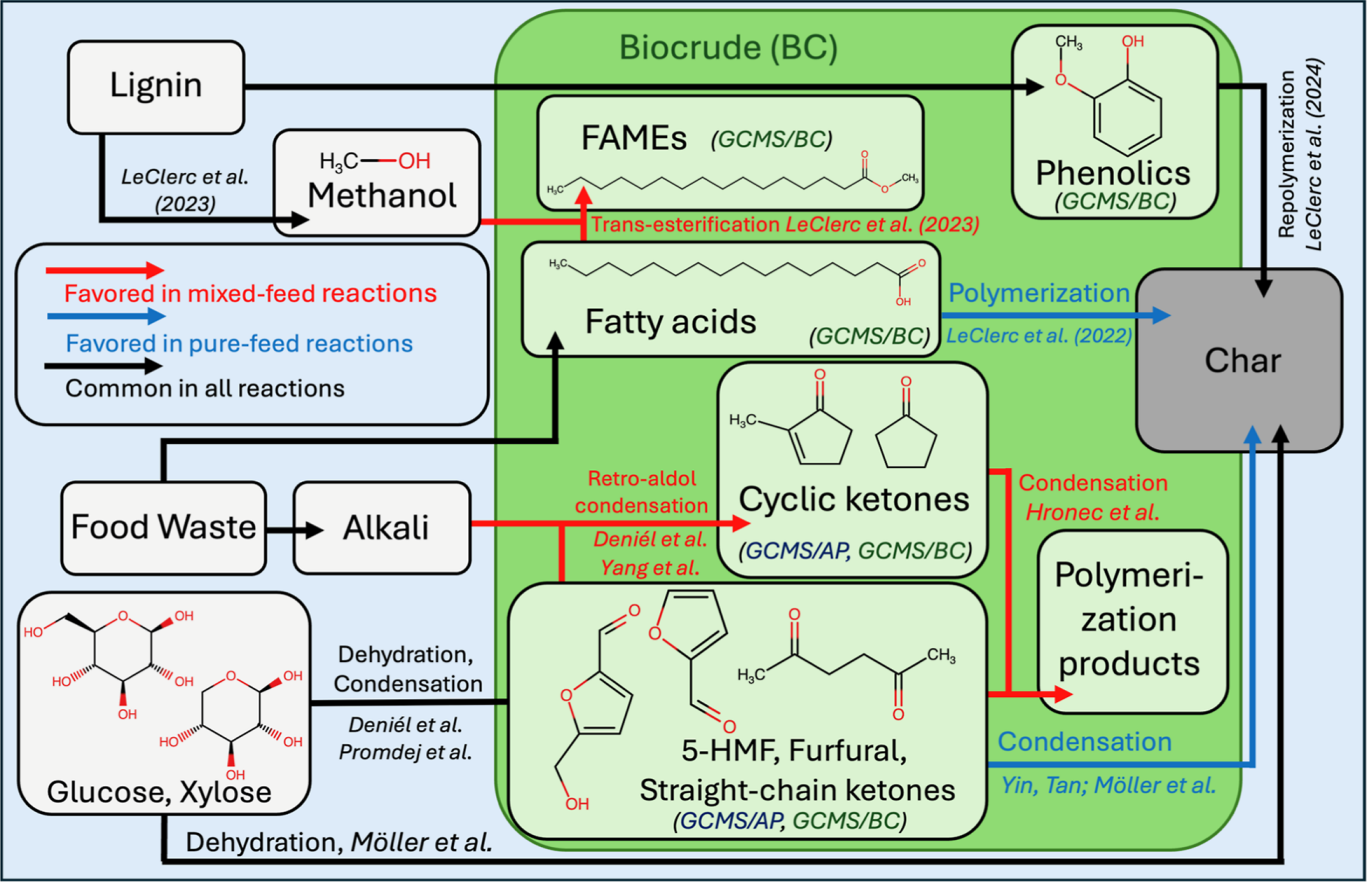
Simplified reaction scheme for co-HTL of food waste with lignocellulose, including pathways most significantly impacted by feedstock interactions.^13,31,33,69,72,92,95,98^

In HTL of cellulose and xylan, the respective glucose and xylose monomer products undergo dehydration and condensation reactions to form reactive aldehydes and straight-chain ketones.^33,95^ In mixed-feed reactions, the alkali content of ash and nitrogen compounds liberated from food waste decomposition catalyzes retro-aldol condensations, sequestering these compounds in the biocrude phase as cyclic ketones and preventing further polymerization of furfural and 5-HMF to char,^69,72^ thus increasing biocrude yields. Accordingly, food waste plays a synergistic role with cellulose and xylan cofeeds by providing an alkali catalyst, as shown in Figure 5. Based on the carbon yields shown previously in Figures 1 and 3, the synergistic catalytic and capping role is more important than the esterification role of cofeed, as indicated by the substantial increase in jet fuel range compounds observed for cofeeding food waste and either cellulose or xylan.

Together, the trans-esterification and alkali catalyzed retro-aldol condensation pathways can explain much of the synergy between biomass model compounds and food waste. However, additional well-known reaction schemes are needed to accurately describe biocrude formation pathways. In all reactions involving food waste, protein degradation products are likely coupled with model compound or food waste derived carbohydrates to form biocrude-range compounds via Maillard reactions.^13,33,96^ While previous studies have observed feed ratio dependence for Maillard reactions,^13,97^ arising from the varied ratio of proteins to carbohydrates, the present work indicates that the high carbohydrate content (53.9%) of the food waste allows for full utilization of the proteins (21.7%), even without the addition of cellulose or xylan, mitigating any feed ratio dependence. Sheng et al. reported that maximum Maillard product yield was obtained at a ratio of 3:1 protein to carbohydrate,^97^ similar the ratio present in the food waste used in this study. An additional feed-ratio independent pathway is the degradation of lignin into a wide range of phenolic compounds.^34,35^ Although these compounds do not appear to be active in any significant synergistic pathway, lignin-derived phenolic compounds comprise a significant fraction of biocrudes from lignin HTL and co-HTL.

Figure 7 showcases how the synergistic effects observed in this study contribute to increased biocrude yields observed in co-HTL of food waste and model biomass compounds. Food waste breaks down rapidly to fatty acids and amino acids, cellulose and xylan hydrolyze to their monomer forms, respectively glucose and xylose, and lignin degrades to form phenolic products. In the presence of cofeed, fatty acids transform into more stable esters and amides, preventing their recombination with other molecules that would result in molecular weight growth out of the jet fuel range and into the vacuum residue range.^98^ Meanwhile, the presence of amino acids and carbohydrates promotes Maillard reactions, as previously reported by others.^13,33,96^ Use of model compounds allows discernment of an additional mechanism involving aldol rearrangements and catalyzed by alkali produced by food waste ash and decomposition of food waste nitrogen compounds to produce ammonia.^74,75^ The net result of these interactions is increased jet fuel and gasoline range compounds, with decreased production of vacuum residue range compounds and especially char – observations made clear in Figures 1 and 3.

Applying these lessons to feedstock selection shows that cofeed processes must be designed with knowledge of cofeed effects, not only on biocrude yield but on quality. Judicious selection of feeds can exploit interactions that promote formation of desirable products, especially jet fuel-range molecules. For maximum jet fuel yield, cofeed of biomass rich in cellulose or xylan is especially favorable, which molecular product analysis ascribes to the combination of fatty acids which form esters and amides and the disruption of char forming pathways due to alkali catalysis. Future work can test this new hypothesis of synergistic production of biocrude with favorable properties with feedstocks carefully selected for low lignin content and establish that improved jet fuel yields that can be obtained from the corresponding biocrudes after hydrotreating,^45^ as well as further isolate the effect of food waste ash in co-HTL.

The importance of catalytic pathways on the synergistic interactions observed in this study encourages careful study of the effect of targeted catalyst addition^26,53,69,70,99^ on biocrude properties. In fact, some feedstocks may contain components that will influence synergistic and antagonistic effects, including the ash and extractive content of biomass.^100^ Similarly, lignin composition varies from source to source and extraction disrupts its native structure.^73^ Different sources of lignin may give rise to important interactions, both in biocrude quantity and properties. Experiments with different types of whole biomass, selected to represent a range of ash, extractives, and lignin contents, can provide a more complete understanding of synergistic and antagonistic interactions during food waste and biomass during co-HTL.

## Conclusion

In the present work, co-HTL was performed by mixing a real food waste with one of three model compounds representing the primary fractions of lignocellulosic biomass: cellulose, xylan, or lignin. Co-HTL of food waste with cellulose or xylan exhibited significant synergistic impacts on biocrude yield, achieving maximum yields of 49.5% and 50.2%, respectively, representing synergistic improvements of 9.1% and 10.1%. Improvements in biocrude yields were largely balanced by corresponding decreases in char production. Furthermore, volatility analysis showed that the increases in biocrude yield primarily benefitted the jet fuel fraction for the cellulose and xylan series, where jet fuel carbon yields were approximately double the expected value for 75:25 food waste–cellulose and food waste–xylan blends, indicating that stabilization or capping of molecules in this range occurred. Interactions between food waste and lignin were less pronounced than observed between food waste and the model carbohydrate biopolymers, resulting in nearly linear yield trends for food waste–lignin blends. Molecular analysis revealed several pathways which explain the observed synergistic interactions between food waste and either cellulose or xylan. In all three series, fatty acids from food waste interact with reactive model compound decomposition intermediates to form FAMEs and fatty amides, decreasing biocrude volatility for enhanced production of jet fuel compared with heavier fractions. Further, food waste is a source of alkali catalyst, converting polymerization-prone furfurals and linear ketones to cyclic ketones via retro-aldol condensations and possibly enhancing carbohydrate hydrolysis. Other pathways, such as Maillard reactions, are present but predominantly independent of feed ratio. These findings provide fundamental insight into the co-HTL of food waste and lignocellulose and may be harnessed to increase the yield and volatility of biocrude toward the production of sustainable jet fuel precursors.

## Supplementary Material



## Notes

Detailed feed composition, detailed product yields and compositions, unprocessed TGA, GCMS, and FTIR data, detailed fuel range mass and carbon yields, GCMS compound tables, furanic/cyclic ketone selectivity (PDF)
