# Supplementary material for: Synergistic Interactions During Co-Hydrothermal Liquefaction of Food Waste and Biomass Model Compounds for Increased Sustainable Aviation Fuel Production
Source: ACS Eng Au. 2026 Apr 27;6(3):490–503. doi: 10.1021/acsengineeringau.6c00009 (PMC13281376; doi:10.1021/acsengineeringau.6c00009)
Supplement: Supplementary file 1 [file eg6c00009_si_001.pdf]

Supporting Information for:

Synergistic Interactions during Co-Hydrothermal Liquefaction of Food Waste and Biomass Model Compounds for Increased Sustainable Aviation Fuel Production

Skyler B. Kauffman[†,‡], Heather O. LeClerc[†,§], Alex R. Maag[†], Geoffrey A. Tompsett[†], Jeffrey R. Page[‖], Ioulia A. Valla[‖], Andrew R. Teixeira[†], Michael T. Timko[†,*]

[†]Department of Chemical Engineering, Worcester Polytechnic Institute, Worcester, MA 01609, United States

[‡]Department of Chemical Engineering and Materials Science, University of Minnesota, Minneapolis, MN 55455, United States

[§]Department of Chemical & Biomedical Engineering, University of Maine, Orono, ME 04469, United States

[‖]Department of Chemical and Biomolecular Engineering, University of Connecticut, Storrs, CT 06269, United States

*corresponding author – mttimko@wpi.edu

Keywords. Hydrothermal Liquefaction, Co-Liquefaction, Biomass, Bioenergy, Aviation Fuel, Sustainability

Table SI-1. Biochemical content of the prison kitchen food waste (PKFW) used in this study.

PKFW Composition (Dry wt. %)	
Lipid	18.6
Protein	21.7
Carbohydrate	53.9
Lignin	0
Ash	5.83

Table SI-2. Elemental content of the prison kitchen food waste (PKFW) and green waste model compounds used in this study.

	Composition (wt. %)			
	PKFW (dry)	Cellulose	Xylan	Lignin
C	0.4737	0.427	0.4215	0.6217
H	0.0695	0.065	0.0541	0.0602
N	0.0486	0	0	0.0081
S				0.0253
O	0.4082	0.508	0.5244	0.2847

Table SI-3. Product carbon yields for co-HTL experiments, calculated relative to total feed carbon.

Feedstock	Biocrude Carbon Yield (%)		Char Carbon Yield (%)		Aqueous Carbon Yield (%)		Gas Carbon Yield (%)	
100% Food Waste	49.06	±3.38	15.20	±1.11	21.25	±2.63	3	±1
25% Cellulose	49.54	±1.97	21.92	±0.86	18.01	±4.07	3	±1
50% Cellulose	44.03	±2.30	25.62	±0.15	19.21	±1.29	3	±1
75% Cellulose	31.85	±3.10	38.14	±1.91	18.14	±1.77	2	±1
100% Cellulose	14.52	±0.93	61.73	±2.15	16.87	±7.25	3	±1
100% Food Waste	49.06	±3.38	15.20	±1.11	21.25	±2.63	3	±1
25% Xylan	50.15	±0.49	25.21	±1.11	17.09	±6.30	3	±1
50% Xylan	33.73	±4.00	38.86	±0.21	21.19	±2.20	3	±1
75% Xylan	25.08	±0.33	52.93	±0.35	10.16	±6.90	3	±1
100% Xylan	13.20	±0.73	67.85	±1.35	11.66	±0.08	2	±1
100% Food Waste	49.06	±3.38	15.20	±1.11	21.25	±2.63	3	±1
25% Lignin	44.22	±0.44	32.82	±0.55	16.88	±1.74	2	±1
50% Lignin	34.10	±2.18	47.02	±3.11	12.78	±0.88	2	±1
75% Lignin	27.39	±8.42	58.52	±3.59	9.67	±0.06	1	±1
100% Lignin	16.72	±4.40	71.79	±0.94	5.76	±0.74	1	±1

Table SI-4. Gravimetrically determined mass yields and biocrude elemental composition for co-HTL experiments performed at at 300 °C for 60 minutes. Biocrude mass yields and elemental content include the contribution of moisture.

Feedstock	Biocrude Mass Yield (%)		Char Mass Yield (%)		Aqueous Mass Yield (%)		Gas Mass Yield (%)		Biocrude Elemental C (%)		Biocrude Elemental H (%)		Biocrude Elemental N (%)	
100% Food Waste	4.95	±0.41	1.66	±0.07	88.24	±0.70	0.9	±0.3	73.40	±2.21	10.50	±0.60	3.13	±0.17
25% Cellulose	4.55	±0.45	2.30	±0.10	87.76	±0.99	0.7	±0.3	73.61	±0.17	8.62	±0.30	2.92	±0.13
50% Cellulose	4.06	±0.41	2.59	±0.03	90.79	±0.40	0.7	±0.1	71.92	±0.17	8.43	±0.13	2.28	±0.10
75% Cellulose	3.12	±0.40	3.79	±0.19	86.25	±0.87	0.6	±0.2	70.00	±0.19	8.16	±0.24	1.38	±0.23
100% Cellulose	1.37	±0.18	6.44	±0.14	94.71	±10.86	0.7	±0.2	65.11	±2.45	7.03	±0.39	0.07	±0.01
100% Food Waste	4.95	±0.41	1.66	±0.07	88.24	±0.70	0.9	±0.3	73.40	±2.21	10.50	±0.60	3.13	±0.17
25% Xylan	4.84	±0.32	2.56	±0.17	86.96	±2.13	0.7	±0.1	72.06	±0.63	10.33	±0.17	2.30	±0.25
50% Xylan	2.70	±0.68	3.87	±0.10	88.83	±2.07	0.7	±0.1	71.43	±1.89	9.77	±0.94	2.20	±0.51
75% Xylan	1.89	±1.15	5.22	±0.04	91.76	±0.61	0.7	±0.2	68.57	±0.68	8.20	±0.97	1.10	±0.16
100% Xylan	1.27	±0.15	6.80	±0.15	98.17	±0.35	0.6	±0.1	71.72	±3.67	5.17	±1.17	0.02	±0.03
100% Food Waste	4.95	±0.41	1.66	±0.07	88.24	±0.70	0.9	±0.3	73.40	±2.21	10.50	±0.60	3.13	±0.17
25% Lignin	4.93	±0.38	3.55	±0.06	84.84	±0.42	0.7	±0.1	69.45	±2.76	9.01	±0.27	2.20	±0.31
50% Lignin	3.93	±0.32	5.27	±0.30	84.52	±0.32	0.5	±0.1	70.50	±0.63	8.93	±0.71	1.87	±0.21
75% Lignin	2.97	±0.65	7.24	±0.68	81.57	±0.06	0.5	±0.1	59.73	±3.14	7.87	±0.37	0.83	±0.10
100% Lignin	0.98	±0.14	9.08	±0.05	81.77	±0.77	0.4	±0.1	54.68	±14.20	5.94	±1.53	0.42	±0.15

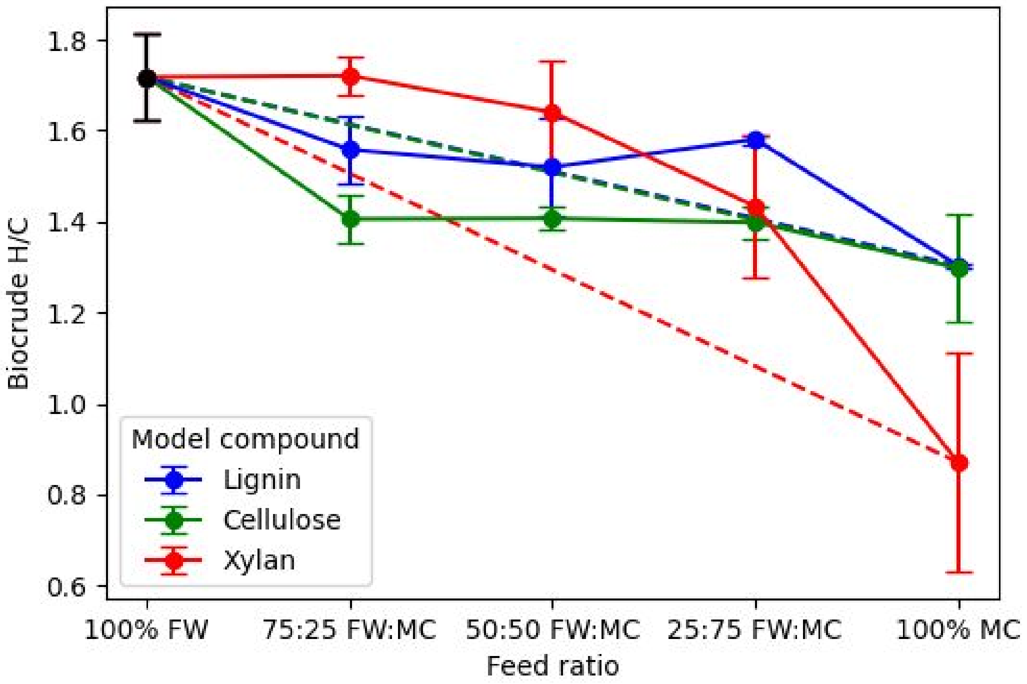

Figure SI-1. Elemental H/C ratio for co-HTL biocrudes obtained by elemental analysis performed on representative samples from co-HTL of various food waste – model compound feed blends reacted at 300 °C for 60 minutes.

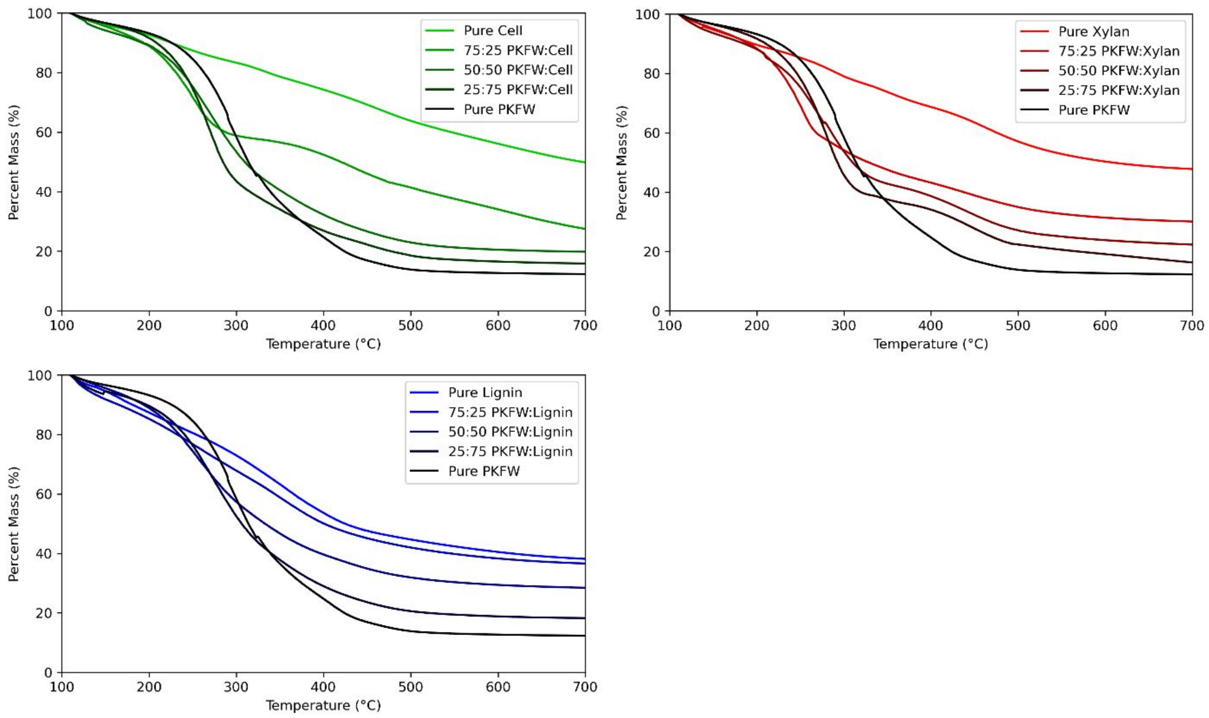

Figure SI-2. Unprocessed mass loss curves obtained from the thermogravimetric analysis (TGA) of biocrude samples using a heating rate of 5 °C/min and a nitrogen flowrate of 25 mL/min.

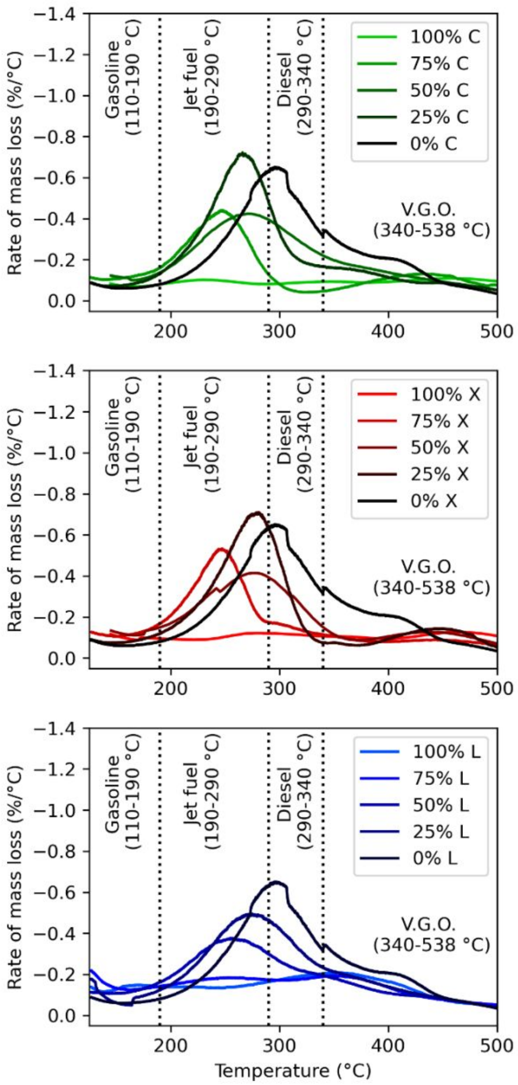

Figure SI-3. Differential thermograms (DTG) for biocrude produced by co-HTL of food waste and the three green waste model compounds. Superimposed fuel ranges are shown for comparison. VGO = vacuum gas oil.

Table SI-5. Mass loss attributed to each temperature range for HTL biocrude samples analyzed via TGA. Temperature ranges are: Gasoline ≤ 190 °C, jet fuel = 190−290 °C, diesel = 290−340 °C, vacuum gas oil = 340−538 °C, residue ≥ 538 °C. Compounds associated with mass loss at T > 538 °C can also be termed asphaltenes.

Feedstock	Gasoline (wt%)	Jet fuel (wt%)	Diesel (wt%)	Vacuum gas oil (wt%)	Residue (wt%)
100% Food Waste	5.9	28.6	25.5	26.5	13.5
25% Cellulose	6.9	45.7	11.6	18.4	17.5
50% Cellulose	9.6	32.3	14.5	20.8	22.8
75% Cellulose	9.4	30.9	2.6	18.6	38.6
100% Cellulose	6.5	9.2	4.4	18.9	61.2
100% Food Waste	5.9	28.6	25.5	26.5	13.5
25% Xylan	7.0	41.3	13.2	17.3	21.2
50% Xylan	10.4	30.7	14.1	18.3	26.6
75% Xylan	9.8	34.2	7.2	15.6	33.2
100% Xylan	8.8	10.0	5.3	21.1	54.7
100% Food Waste	5.9	28.6	25.5	26.5	13.5
25% Lignin	9.1	33.9	17.0	20.4	19.8
50% Lignin	9.3	30.4	11.6	17.9	30.8
75% Lignin	13.3	17.1	8.8	20.5	40.3
100% Lignin	11.2	14.1	9.3	22.4	43.0

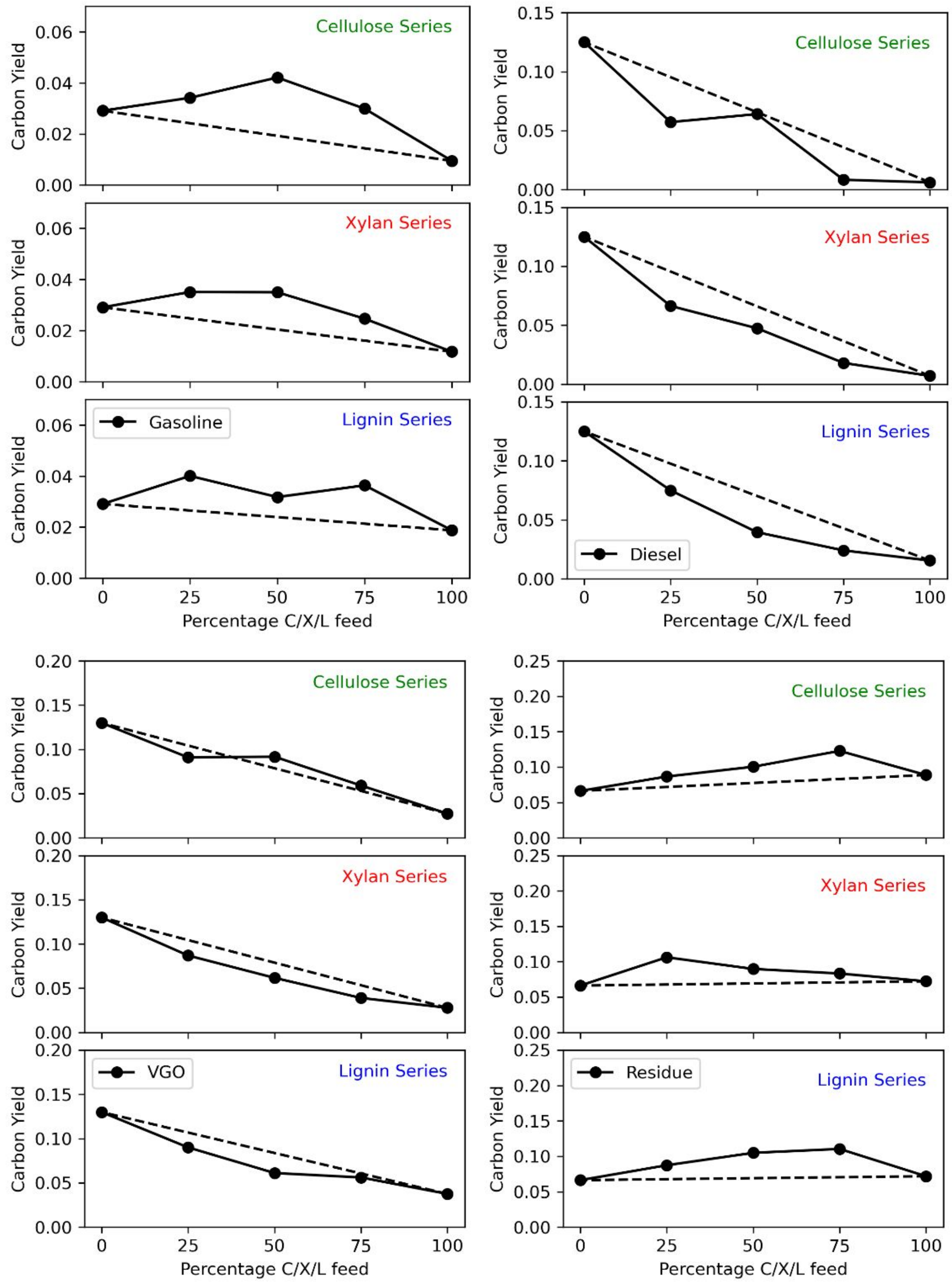

Figure SI-4. Carbon yield-weighted simulated fuel ranges for the various co-HTL biocrudes, calculated relative to total feed carbon.

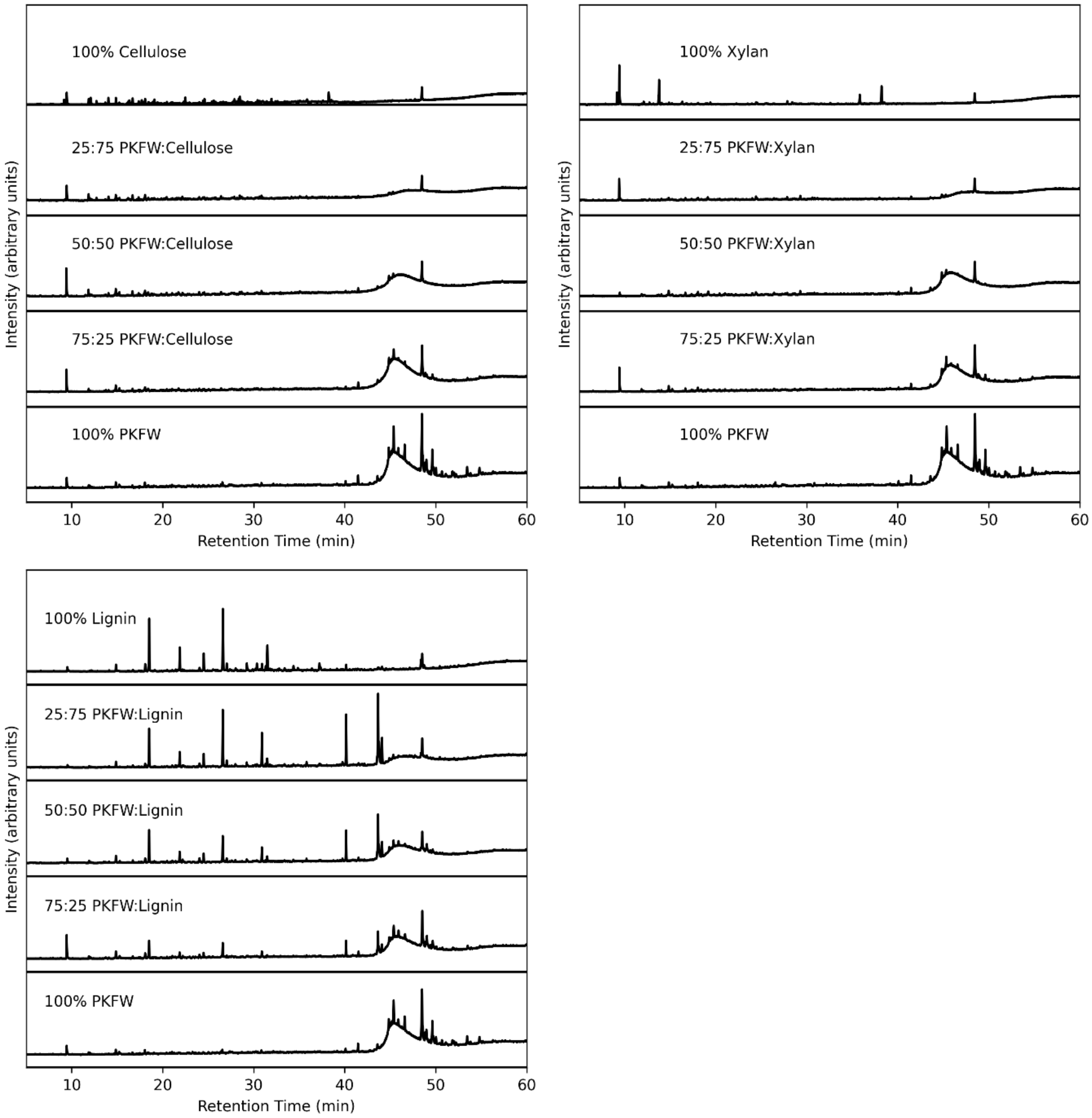

Figure SI-5. Unprocessed GC-MS chromatograms obtained from the analysis of co-HTL biocrudes.

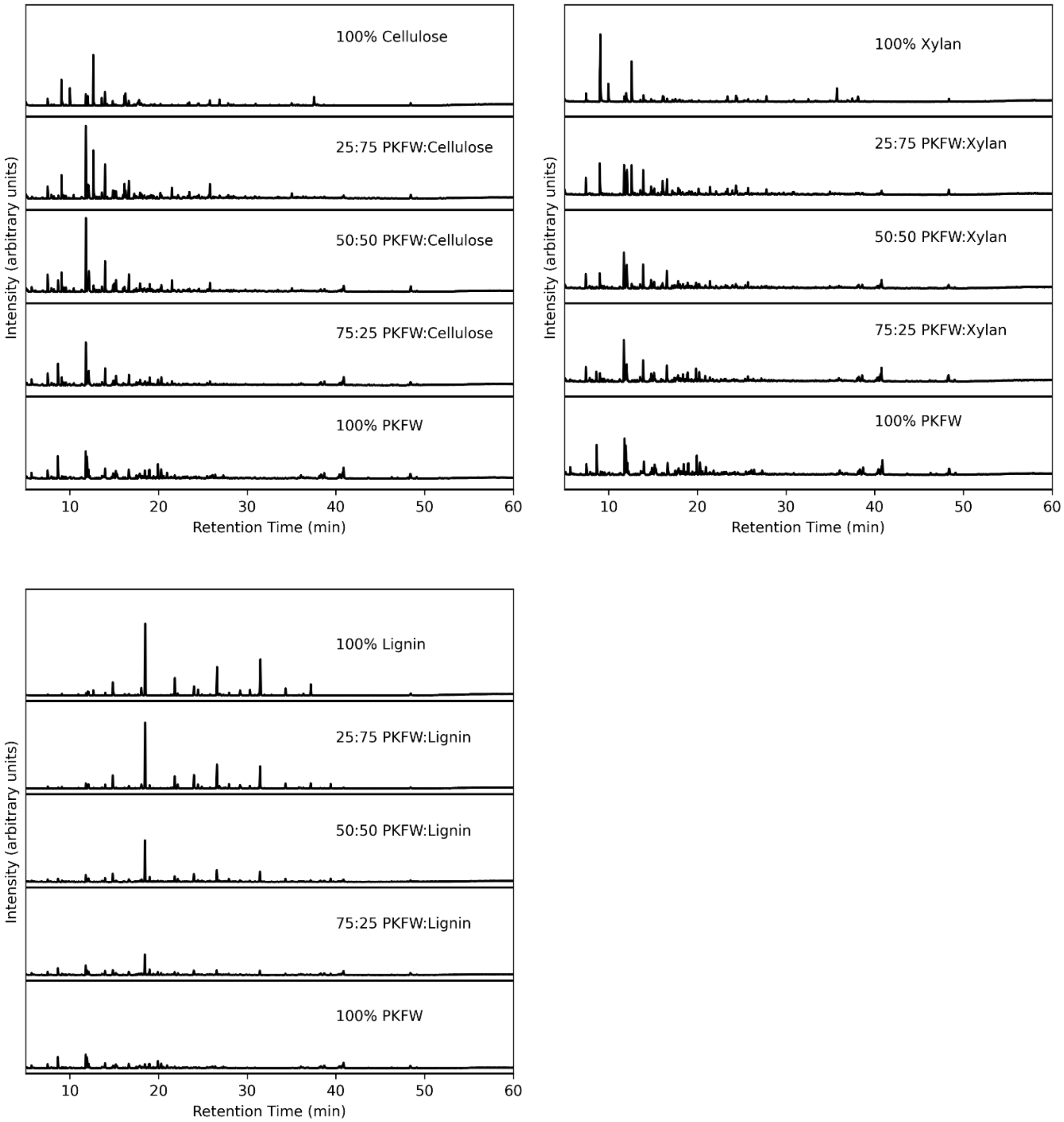

Figure SI-6. Unprocessed GC-MS chromatograms obtained from the analysis of co-HTL aqueous phase. Aqueous phase was pre-separated followed by acidification and separation with DCM as explained in the Methods section.

Table SI-6. List of all GC retention times and mass spectrometer assignments for co-HTL biocrudes.

Retention Time	Compound Name	Category
	**Lignin-Food Waste Biocrudes**	
9.444	2-Pentanone, 4-hydroxy-4-methyl-	Acetone
14.872	Phenol	Phenol
18.075	Phenol, 3-methyl-	Phenol
18.08	p-Cresol	Aromatic
18.519	Phenol, 2-methoxy-	Phenol
21.879	Creosol	Aromatic
24.047	1,2-Benzenediol, 3-methoxy-	Aromatic
24.505	Phenol, 4-ethyl-2-methoxy-	Phenol
26.617	Phenol, 2-(1,1-dimethylethyl)-4-methyl-	Phenol
27.052	Phenol, 2-methoxy-4-propyl-	Phenol
29.246	1,2,4-Trimethoxybenzene	Aromatic
30.356	Apocynin	Aromatic
30.912	Butylated Hydroxytoluene	Aromatic
31.325	Benzene, 1,2,3-trimethoxy-5-methyl-	Aromatic
31.503	2-Propanone, 1-(4-hydroxy-3-methoxyphenyl)-	Acetone
34.375	Methyl-(2-hydoxy-3-ethoxy-benzyl)ether	Aromatic
37.222	Desaspidinol	Aromatic
40.155	Hexadecanoic acid, methyl ester	FAME
41.519	Hexadecanoic acid, ethyl ester	FAME
43.567	Octadecanoic acid	Fatty Acid
43.568	9,12-Octadecadienoic acid (Z,Z)-, methyl ester	FAME
43.662	11-Octadecenoic acid, methyl ester	FAME
44.096	Tetradecanoic acid, 12-methyl, methyl ester	FAME
44.099	Methyl stearate	FAME
44.895	Ethyl Oleate	FAME
44.898	(E)-9-Octadecenoic acid ethyl ester	FAME
44.972	9-Octadecenoic acid, ethyl ester	FAME
45.03	Pentadecanoic acid	Fatty Acid
45.22	2-Oxepanone, 7-butyl-	Other
45.344	Octadecanamide	Amide
45.363	Hexadecanoic acid	Fatty Acid

Retention Time	Compound Name	Category
45.5	Carbonic acid, nonyl 2,2,2-trichloroethyl ester	Other
45.54	11-Bromoundecanoic acid	Fatty Acid
45.55	4-Hexadecanol	Fatty Acid
45.66	Ascorbyl Palmitate	Other
45.91	N-Methyldodecanamide	Amide
45.918	Eicosanoic Acid	Fatty Acid
46.12	Cyclopentadecanone, 2-hydroxy-	Other
46.13	Cyclopentanepropanoic acid, 1-acetyl-2,2-dimethyl-, methyl ester	FAME
46.28	Azacyclotridecan-2-one	Other
46.599	N,N-Dimethyldodecanamide	Amide
46.63	Dodecanoic acid, 2-octyl	Fatty Acid
46.74	Acetamide, N,N'-1,4-butanediylbis-	Amide
47.09	Tetradecanamide	Amide
47.52	Valeraldehyde, semicarbazone	Other
47.68	1H-Imidazole-1-ethanol, 2-heptadecyl-4,5-dihydro-	Other
47.87	2-Propenoic acid, 2-methyl-, cyclohexyl ester	FAME
48.172	Acetoxyacetic acid, 5-tetradecyl ester	FAME
48.378	Ethyl homovanillate	Aromatic
48.381	(-)-Nortrachelogenin	Aromatic
48.514	9-Octadecenamide, (Z)-	Amide
48.714	Homovanillyl alcohol	Aromatic
48.906	Hexadecanamide	Amide
49.636	9-Octadecenamide, N,N-dimethyl-	Amide
53.459	Oleic diethanolamide	Amide
61.694	dl-.alpha.-Tocopherol	Other
**Xylan-Food Waste Biocrudes**		
9.154	Furfural	Furfural
9.39	2-Pentanone, 4-hydroxy-4-methyl-	Other Oxygenate
12.083	Ethanone, 1-(2-furanyl)-	Furfural
13.715	3-Aminopyrazine 1-oxide	Other Oxygenate
13.78	2-Furethanol, beta-methoxy	Furfural
14.831	Phenol	Phenol
16.33	2-Heptanone, 1-ethoxy-	Other Oxygenate
16.661	2-Cyclopenten-1-one, 2,3-dimethyl-	Other Oxygenate
16.668	2-Cyclopenten-1-one, 2,3-dimethyl-	Furfural

Retention Time	Compound Name	Category
18.043	p-Cresol	Aromatic
18.053	Phenol, 3-methyl-	Phenol
19.134	3-Pyridinol	Amide
19.41	3-Hydroxydecanoic acid	Fatty Acid
24.004	Benzenemethanol, 4-hydroxy-	Other Oxygenate
24.01	1,2-Benzenediol, 3-methyl-	Other Oxygenate
24.425	Ethanone, 1-(2,5-dihydroxyphenyl)-	Other Oxygenate
24.437	Resorcinol, 2-acetyl-	Phenol
27.846	Ethanone, 1-(3-hydroxyphenyl)-	Other Oxygenate
27.866	Furan, 2,2'-methylenebis-	Furfural
29.295	1H-Inden-1-one, 2,3-dihydro-7-hydroxy-3-methyl-	Phenol
30.616	2H-1-Benzopyran-2-one, 3,4-dihydro-6-methyl-	Other Oxygenate
35.834	1,6(2H,7H)-Naphthalenedione, 3,4,8,8a-tetrahydro-8a-methyl-	Phenol
37.98	1(2H)-Naphthalenone, 3,4-dihydro-6,7-dihydroxy-	Other Oxygenate
38.23	1,8-Naphthalenedione, 8a-ethylperhydro	Other Oxygenate
41.465	Hexadecanoic acid, ethyl ester	FAME
43.595	11-Octadecenoic acid, methyl ester, (Z)-	FAME
44.62	6-Bromohexanoic acid, decyl ester	FAME
44.765	.gamma.-Gurjunenepoxide-(1)	Other Oxygenate
44.842	Eicosanoic acid	Fatty Acid
44.846	(E)-9-Octadecenoic acid ethyl ester	FAME
44.854	Ethyl Oleate	FAME
44.955	Levo-menthoxyacetic acid	Other Oxygenate
44.96	Oleic acid, butyl ester	Fatty Acid
44.972	9-Octadecenoic acid, ethyl ester	FAME
44.98	Succinic acid, 3,7-dimethyloct-6-en-1-yl octadecyl ester	FAME
44.988	13-Docosenamide, (Z)-	Amide
45	2-Formyl-4-methylpentanoic acid, ethyl ester	FAME
45.025	Pentadecanoic acid	Fatty Acid
45.11	4-Dodecanol	Fatty Acid

Retention Time	Compound Name	Category
45.2	.alpha.-D-Glucopyranoside, methyl 4,6-O-nonylidene-	Other Oxygenate
45.281	Butanedioic acid, 2,3-dimethyl-, dimethyl ester	FAME
45.333	Octadecanamide	Amide
45.38	Hexadecanoic acid	Fatty Acid
45.45	Cyclooctancarboxylic acid, 4-methyl-, ethyl ester	FAME
45.51	16-Hydroxyhexadecanoic acid	Fatty Acid
45.54	11-Bromoundecanoic acid	Fatty Acid
45.57	7-Methyl-trans-8-thiabicyclo[4.3.0]nonane	Other Oxygenate
45.66	Z-13,13-Dimethyl-10-tetradecen-1-ol	Fatty Acid
45.696	Adipic acid, di(2,2-dichloroethyl) ester	Other Oxygenate
45.805	n-Hexadecanoic acid	Fatty Acid
45.866	N-Methyldodecanamide	Amide
45.971	[1,1'-Bicyclopropyl]-2-octanoic acid, 2'-hexyl-, methyl ester	FAME
46.12	Cyclopentadecanone, 2-hydroxy-	Other
46.28	Azacyclotridecan-2-one	Other
46.582	N,N-Dimethyldecanamide	Amide
46.74	Acetamide, N,N'-1,4-butanediylbis-	Amide
46.97	Dodecanoic acid, 2-octyl-	Fatty Acid
47.09	Tetradecanamide	Amide
47.52	Valeraldehyde, semicarbazone	Other
47.68	1H-Imidazole-1-ethanol, 2-heptadecyl-4,5-dihydro-	Other
47.87	2-Propenoic acid, 2-methyl-, cyclohexyl ester	FAME
48.172	Acetoxyacetic acid, 5-tetradecyl ester	FAME
48.462	9-Octadecenamide, (Z)-	Amide
49.624	9-Octadecenamide, N,N-dimethyl-	Amide
53.459	Oleic diethanolamide	Amide
**Cellulose-Food Waste Biocrudes**		
9.16	2-Cyclopenten-1-one	Other Oxygenate
9.447	2-Pentanone, 4-hydroxy-4-methyl-	Other Oxygenate
11.868	2-Cyclopenten-1-one, 2-methyl-	Other Oxygenate

Retention Time	Compound Name	Category
12.089	Ethanone, 1-(2-furanyl)-	Furfural
12.722	2,5-Hexanedione	Other Oxygenate
13.736	Pentanoic acid, 4-methyl-	Other Oxygenate
14.03	2-Cyclopenten-1-one, 3-methyl-	Other Oxygenate
14.855	Phenol	Phenol
16.335	2-Cyclopenten-1-one, 2-hydroxy-3-methyl-	Other Oxygenate
16.693	2-Cyclopenten-1-one, 2,3-dimethyl-	Other Oxygenate
17.366	Phenol, 2-methyl-	Phenol
17.691	Pentanoic acid, 4-oxo-	Other Oxygenate
17.95	2-Cyclopenten-1-one, 3-ethyl-	Other Oxygenate
18.054	p-Cresol	Phenol
18.076	Phenol, 3-methyl-	Phenol
18.393	Cyclohexanone, 3-ethenyl-	Other Oxygenate
18.411	Cyclohexane, (1-methylethylidene)-	Other Oxygenate
19.096	Benzofuran, 2-methyl-	Furfural
20.436	Phenol, 2,5-dimethyl-	Phenol
20.44	Phenol, 2,6-dimethyl-	Phenol
21.731	3-Cyclohexen-1-one, 3,5,5-trimethyl-	Other Oxygenate
22.152	Catechol	Phenol
22.49	Benzofuran, 4,7-dimethyl-	Furfural
24.012	9-(S)-Methyl-.delta.-5(10)-octalin-1,6-dione	Other Oxygenate
24.014	Benzenemethanol, 4-hydroxy-	Other Oxygenate
24.452	Resorcinol, 2-acetyl-	Phenol
24.593	1H-Inden-1-one, 2,3-dihydro-	Phenol
25.605	2-Coumaranone	Other Oxygenate
25.823	2-Hexenoic acid, 3,4,4-trimethyl-5-oxo-, (Z)-	Fatty Acid
26.403	1,4-Benzenediol, 2-methyl-	Other Oxygenate
27.882	Ethanone, 1-(3-hydroxyphenyl)-	Other Oxygenate
28.351	7-Methylindan-1-one	Other Oxygenate
28.486	2-Methyl-5-hydroxybenzofuran	Furfural
28.669	2-Methoxy-5-methylphenol	Phenol
30.412	4-Hydroxy-2-methylacetophenone	Other Oxygenate
30.645	1,4-Benzenedicarboxaldehyde, 2,5-dimethyl-	Other Oxygenate

Retention Time	Compound Name	Category
30.858	Ethanone, 1-(2,5-dihydroxyphenyl)-	Other Oxygenate
31.944	1-Naphthalenol, 4-methoxy-	Other Oxygenate
34.145	Anisole, o-(1-ethylvinyl)-	Phenol
35.804	Indene-1,7(4H)-dione, 3a,7a-dihydro-5-methyl-	Phenol
35.854	1,6(2H,7H)-Naphthalenedione, 3,4,8,8a-tetrahydro-8a-methyl-	Phenol
38.245	2H-1-Benzopyran-2-one, 3,5,7-trihydroxy-	Other Oxygenate
38.525	1H-Isoindole-1,3(2H)-dione, 2-[4-[4,4-dimethyl-2-(methylamino)-6-oxo-1-cyclohexenyl]-4-oxobutyl]-	Other Oxygenate
41.484	Hexadecanoic acid, ethyl ester	FAME
43.619	9-Octadecenoic acid, methyl ester, (E)-	FAME
43.622	11-Octadecenoic acid, methyl ester, (Z)-	FAME
44.66	n-Butyl laurate	FAME
44.785	12-Methyl-E,E-2,13-octadecadien-1-ol	Other Oxygenate
44.862	(E)-9-Octadecenoic acid ethyl ester	FAME
44.865	Ethyl Oleate	FAME
44.99	(2S,2'S)-2,2'-Bis[1,4,7,10-tetraoxacyclododecane]	Other Oxygenate
45.001	13-Docosenamide, (Z)-	Amide
45.03	Undecanol-4	Fatty Acid
45.13	6-(1-Hydroxyethyl)-2-iodo-4-oxa-tricyclo[4.2.1.0(3,7)]nonan-5-one	Other Oxygenate
45.141	Dimethylmalonic acid, 3-ethylphenyl pentyl ester	FAME
45.26	Nonanedioic acid, dimethyl ester	FAME
45.296	Dodecanamide	Amide
45.32	Ethyl tridecanoate	FAME
45.339	Octadecanamide	Amide
45.386	Hexadecanamide	Amide
45.51	Pentanedioic acid, 2-methyl-, bis(1-methylpropyl) ester	FAME
45.545	3-cis-Methoxy-5-trans-methyl-1R-cyclohexanol	Other Oxygenate

Retention Time	Compound Name	Category
45.625	Hexanedioic acid, 3-oxo-, diethyl ester	FAME
45.733	E-12-Tetradecen-1-ol acetate	Fatty Acid
45.83	Benzo[b]thiophene, octahydro-2-methyl (2.alpha., 3a.beta., 7a.beta.)-	Other Oxygenate
45.895	2-Nonenoic acid	Fatty Acid
45.925	l-(+)-Ascorbic acid 2,6-dihexadecanoate	Other Oxygenate
48.484	9-Octadecenamide, (Z)-	Amide
48.57	Cyclopentanecarboxylic acid, 2-tridecyl ester	FAME
49.648	9-Octadecenamide, N,N-dimethyl-	Amide

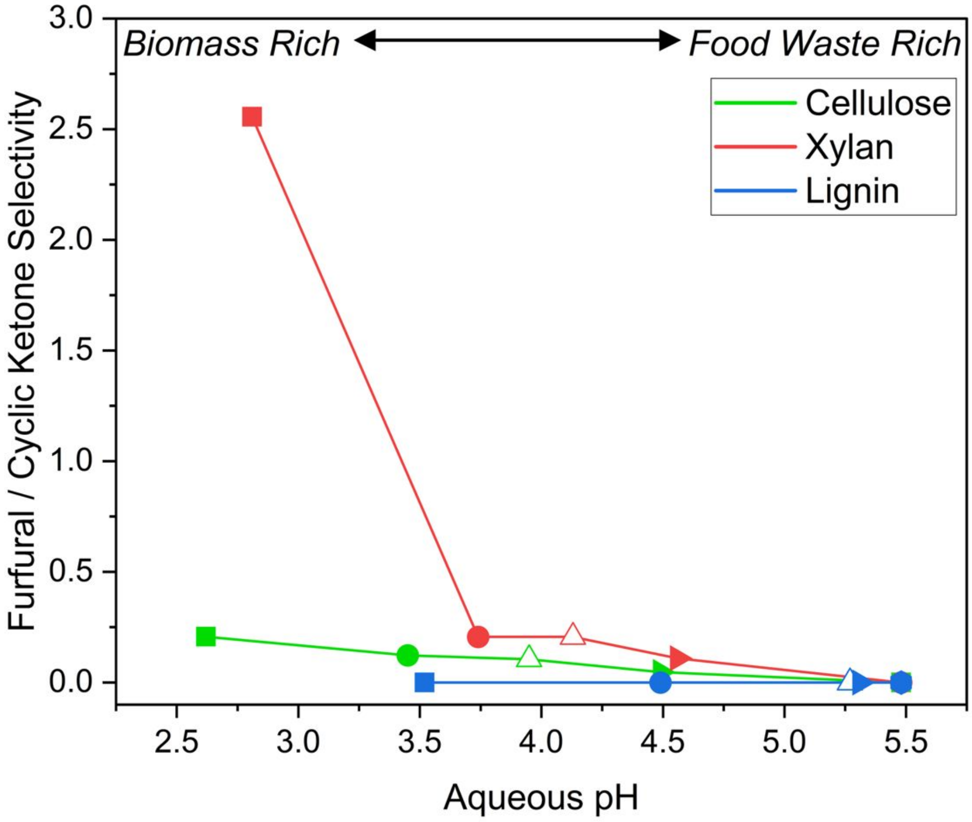

Figure SI-7. Selectivity of cyclic ketone compounds over furanic (primarily furfural) compounds in co-HTL aqueous phase as a function of aqueous pH. Selectivity determined from GC-MS analysis of aqueous phase extracts and taken as a ratio of the total relative area. Symbol shapes represent the percentage of food waste in the feed, where square = 0% food waste, circle = 25% food waste, open triangle = 50% food waste, right facing triangle = 75% food waste, and hexagon = 100% food waste.

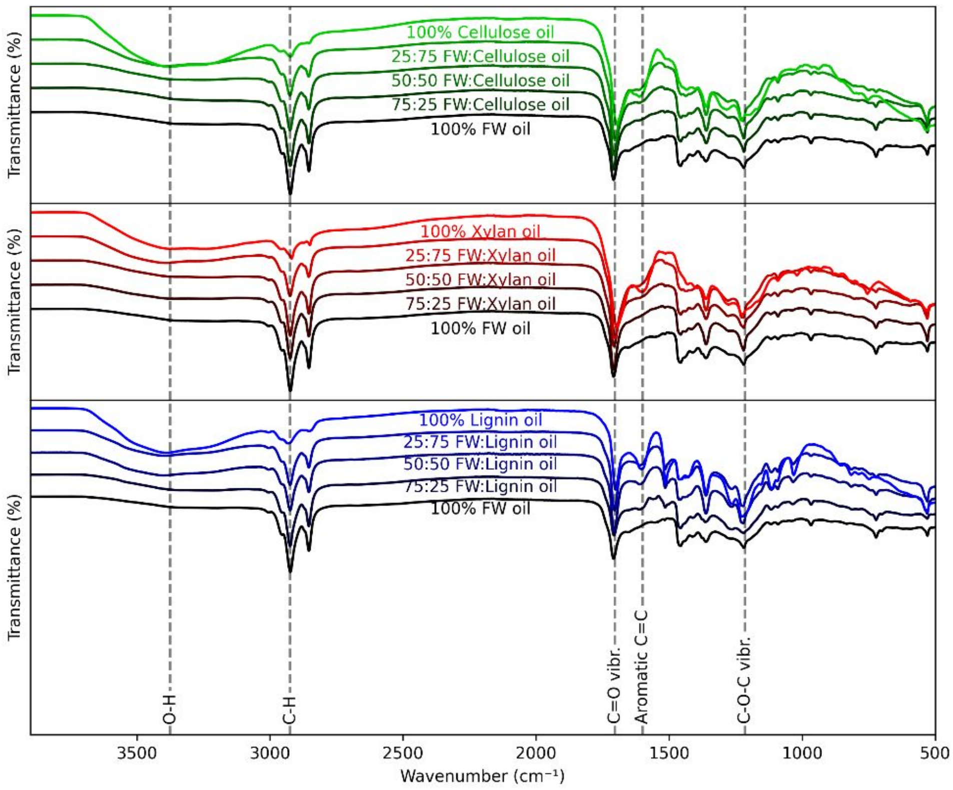

Figure SI-8. Unprocessed, offset FT-IR spectra for all co-HTL biocrudes.

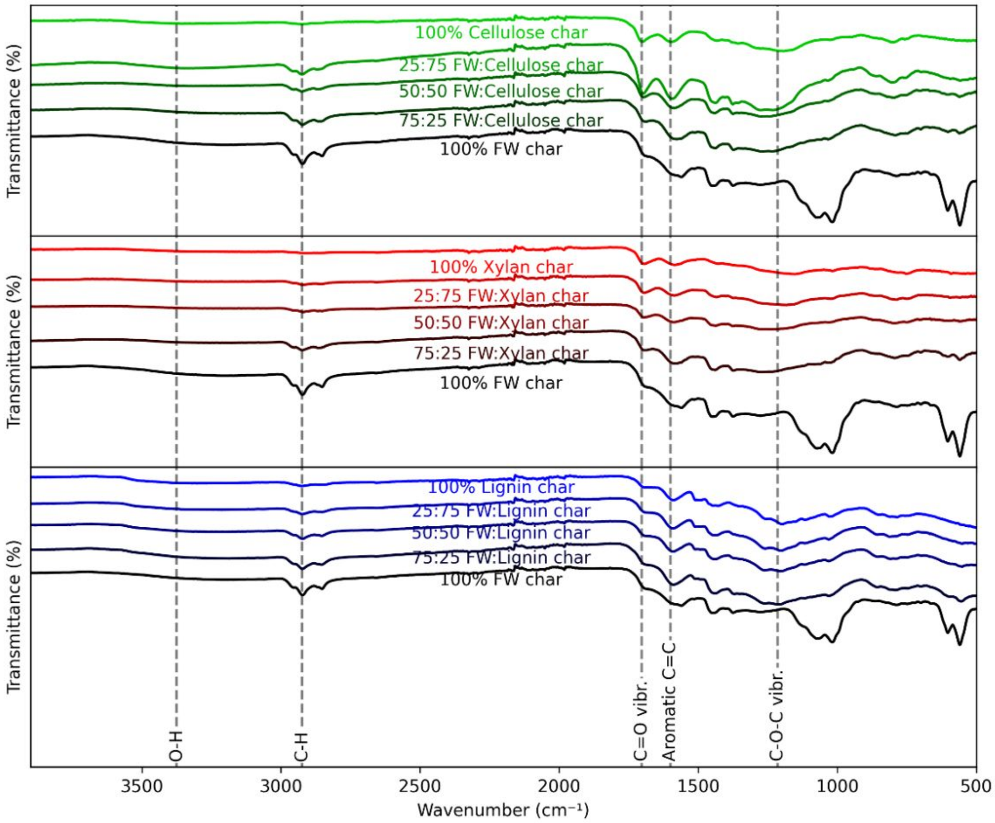

Figure SI-9. Unprocessed, offset FT-IR spectra for all co-HTL chars.
